# Automatic Filtering and Substantiation of Drug Safety Signals

**DOI:** 10.1371/journal.pcbi.1002457

**Published:** 2012-04-05

**Authors:** Anna Bauer-Mehren, Erik M. van Mullingen, Paul Avillach, María del Carmen Carrascosa, Ricard Garcia-Serna, Janet Piñero, Bharat Singh, Pedro Lopes, José L. Oliveira, Gayo Diallo, Ernst Ahlberg Helgee, Scott Boyer, Jordi Mestres, Ferran Sanz, Jan A. Kors, Laura I. Furlong

**Affiliations:** 1 Research Programme on Biomedical Informatics (GRIB), IMIM-Hospital del Mar Research Institute, DCEX, Universitat Pompeu Fabra, Barcelona, Spain; 2 Erasmus University Medical Center, Rotterdam, The Netherlands; 3 LESIM-ISPED, Université de Bordeaux, Bordeaux, France; 4 LERTIM, EA 3283, Faculté de Médecine, Université de Aix-Marseille, Marseille, France; 5 DETI/IEETA, Universidade de Aveiro, Aveiro, Portugal; 6 AstraZeneca, Mölndal, Sweden; Stanford University, United States of America

## Abstract

Drug safety issues pose serious health threats to the population and constitute a major cause of mortality worldwide. Due to the prominent implications to both public health and the pharmaceutical industry, it is of great importance to unravel the molecular mechanisms by which an adverse drug reaction can be potentially elicited. These mechanisms can be investigated by placing the pharmaco-epidemiologically detected adverse drug reaction in an information-rich context and by exploiting all currently available biomedical knowledge to substantiate it. We present a computational framework for the biological annotation of potential adverse drug reactions. First, the proposed framework investigates previous evidences on the drug-event association in the context of biomedical literature (signal filtering). Then, it seeks to provide a biological explanation (signal substantiation) by exploring mechanistic connections that might explain why a drug produces a specific adverse reaction. The mechanistic connections include the activity of the drug, related compounds and drug metabolites on protein targets, the association of protein targets to clinical events, and the annotation of proteins (both protein targets and proteins associated with clinical events) to biological pathways. Hence, the workflows for signal filtering and substantiation integrate modules for literature and database mining, *in silico* drug-target profiling, and analyses based on gene-disease networks and biological pathways. Application examples of these workflows carried out on selected cases of drug safety signals are discussed. The methodology and workflows presented offer a novel approach to explore the molecular mechanisms underlying adverse drug reactions.

## Author Summary

Adverse drug reactions (ADRs) constitute a major cause of morbidity and mortality worldwide. Due to the relevance of ADRs for both public health and pharmaceutical industry, it is important to develop efficient ways to monitor ADRs in the population. In addition, it is also essential to comprehend why a drug produces an adverse effect. To unravel the molecular mechanisms of ADRs, it is necessary to consider the ADR in the context of current biomedical knowledge that might explain it. Nowadays there are plenty of information sources that can be exploited in order to accomplish this goal. Nevertheless, the fragmentation of information and, more importantly, the diverse knowledge domains that need to be traversed, pose challenges to the task of exploring the molecular mechanisms of ADRs. We present a novel computational framework to aid in the collection and exploration of evidences that support the causal inference of ADRs detected by mining clinical records. This framework was implemented as publicly available tools integrating state-of-the-art bioinformatics methods for the analysis of drugs, targets, biological processes and clinical events. The availability of such tools for *in silico* experiments will facilitate research on the mechanisms that underlie ADR, contributing to the development of safer drugs.

## Introduction

Drug safety issues can arise during pre-clinical screening, clinical trials and, more importantly, after the drug is marketed and tested for the first time on the population [1]. Although relatively rare once a drug is marketed, drug safety issues constitute a major cause of morbidity and mortality worldwide.

In 1998, Lazarou et al estimated that yearly about 2 million patients in the US are affected by a serious adverse drug reactions (ADRs) resulting in approximately 100 000 fatalities, ranking ADRs between the fourth and sixth cause of death in the US, not far behind cancer and heart diseases [2]. Similar figures were estimated more recently for other western countries [3], [4], [5]. Serious ADRs resulting from the treatment with thalidomide prompted modern drug legislation more than 40 years ago [6]. Over the past 10 years, 19 broadly used marketed drugs were withdrawn after presenting unexpected side effects [1], [3]. The current and future challenges of drug development and drug utilization, and a number of recent high-impact drug safety issues (e.g. rofecoxib) highlight the need of an improvement of safety monitoring systems [5]. In this regard, initiatives such as the EC-funded EU-ADR project seek to develop methodologies to improve the way drug safety signals are detected and analyzed [7], [8].

Due to the important implications of an ADR in both public health and the pharmaceutical industry, unraveling the molecular mechanisms by which the ADR is elicited is of great relevance. Understanding the molecular mechanisms of ADRs can be achieved by placing the drug adverse reaction in the context of current biomedical knowledge that might explain it. Due to the huge amounts of data generated by the “omics” experiments, and the ever-increasing volume of data and knowledge stored in databases related with ADRs, the application of bioinformatics analysis tools is essential in order to study and analyze the molecular and biological basis of ADRs.

### ADR mechanisms

Although the factors that determine the susceptibility to ADRs are not completely well understood, accumulating evidence over the years indicate an important role of genetic factors [9]. ADRs can be mechanistically related to drug metabolism phenomena, leading for instance to an unusual drug accumulation in the body [9]. They can be associated with inter-individual genetic variants, most notably single nucleotide polymorphisms (SNPs), in genes encoding drug metabolizing enzymes and drug target genes [9]. One of the first ADRs explained by a genetic factor was the inherited deficiency of the enzyme glucose-6-phosphate dehydrogenase causing severe anemia in patients treated with the antimalarial drug primaquine [10]. Alternatively, an ADR can be caused by the interaction of the drug with a target different from the originally intended target (also known as off-targets) [11]. A well-known example of an off-target ADR is provided by aspirin, whose anti-inflammatory effect, exerted by inhibition of prostaglandin production by COX-2, comes at the expense of irritation of the stomach mucosa by its unintended inhibition of COX-1 [12], [13]. Furthermore, in addition to mechanisms related to off-target pharmacology, it is becoming evident that ADRs may often be caused by the combined action of multiple genes [9]. The anticoagulant warfarin, which shows a varying degree of anticoagulant effects, is often associated with hemorrhages, and leads the list of drugs with serious ADR in the US and Europe [9]. A 50% of the variable effects of warfarin are explained by polymorphisms in the genes CYP2C9 and VKORC1 [14], [15]. A recent study furthermore identified a third gene, CYP4F2 explaining about 1.5% of dose variance [16]. However, the genes accounting for the remaining variability in the response to warfarin are still unknown.

Other cases of ADRs may arise as a consequence of drug-drug interactions, or the interplay between the effect of the drug and environmental factors [9], [15]. Indeed, the interaction between genotype and environment observed in several aspects of health and disease also extend to drug response and safety. For example, alcohol consumption and smoking are both associated with changes in the expression of the metabolic enzyme CYP2E1, therefore affecting the pharmacokinetics of certain drugs [17].

### Challenges in studying ADRs

From the above paragraphs, it is clear that the study of the molecular and biological mechanisms underlying ADRs requires achieving a synthesis of information across multiple disciplines. In particular, it requires the integration of information from a variety of knowledge domains, ranging from the chemical to the biological up to the clinical. Different resources cover information about these different knowledge domains, and many of them are freely available on the web, such as biological and chemical databases and the biomedical literature. On the other side, new data is produced continuously, and the list of resources and published papers that a researcher interested in ADRs needs to cope with is turning more into a problem than into a solution. It has been recognized that the adequate management of knowledge is becoming a key factor for biomedical research, especially in the areas that require traversing different disciplines and/or the integration of diverse and heterogeneous pieces of information [18]. A key aspect is the integration of heterogeneous data types, and several authors have discussed the challenges of data integration in the life sciences [19], [20], which are rooted in the inherent complexity of the biological domain, its high degree of fragmentation, the data deluge problem, and the widespread ambiguity in the naming of entities [21]. In addition to the complexity of extracting, storing and integrating heterogeneous data from multiple domains one needs to consider the lack of completeness of the data available [22], an aspect that has a direct impact on the scope and conclusions of any analysis performed on the integrated data.

On the other hand, approaching current biomedical research questions by computational analysis requires a combination of different methods. An attractive approach that emerged in the last years is the combination of different bioinformatics analysis modules by means of pipelines or workflows [23]. This technology allows the integration of a variety of computational techniques into a processing pipeline in which the input and outputs are standardized. This kind of integration has been greatly facilitated by the use of public APIs and web services allowing programmatic access to data repositories and analysis tools. The open source software Taverna is one of such approaches that allow integration of different analysis modules, shared as web services, into a scientific workflow to perform *in silico* experiments [24]. Similar approaches are also used for the processing of free-text documents (http://uima.apache.org/) or for combining data mining methods (http://www.knime.org/).

In this article we present a general framework developed in the context of the EU-ADR project for a systematic analysis of adverse drug reactions. The entry point of the system is a potential drug safety signal, which is composed of the drug and its associated adverse reaction. In the process of *signal filtering*, we search for previous reports of the potential signal in specialized databases and in the biomedical literature. In the process of *signal substantiation*, we seek to provide a plausible biological explanation to the potential signal. This framework was implemented by means of software modules accessible through web services and integrated into workflows ready to be used for automatic filtering and substantiation of drug-event associations. Finally, we present a detailed analysis of antipsychotic drugs and their association with the prolongation of the QT interval, as well as a large scale analysis of drug-side effect pairs from SIDER [25] emphasizing the usefulness of our signal filtering and substantiation workflows.

## Results

### A framework for the filtering and substantiation of drug-event pairs

**Figure 1:**
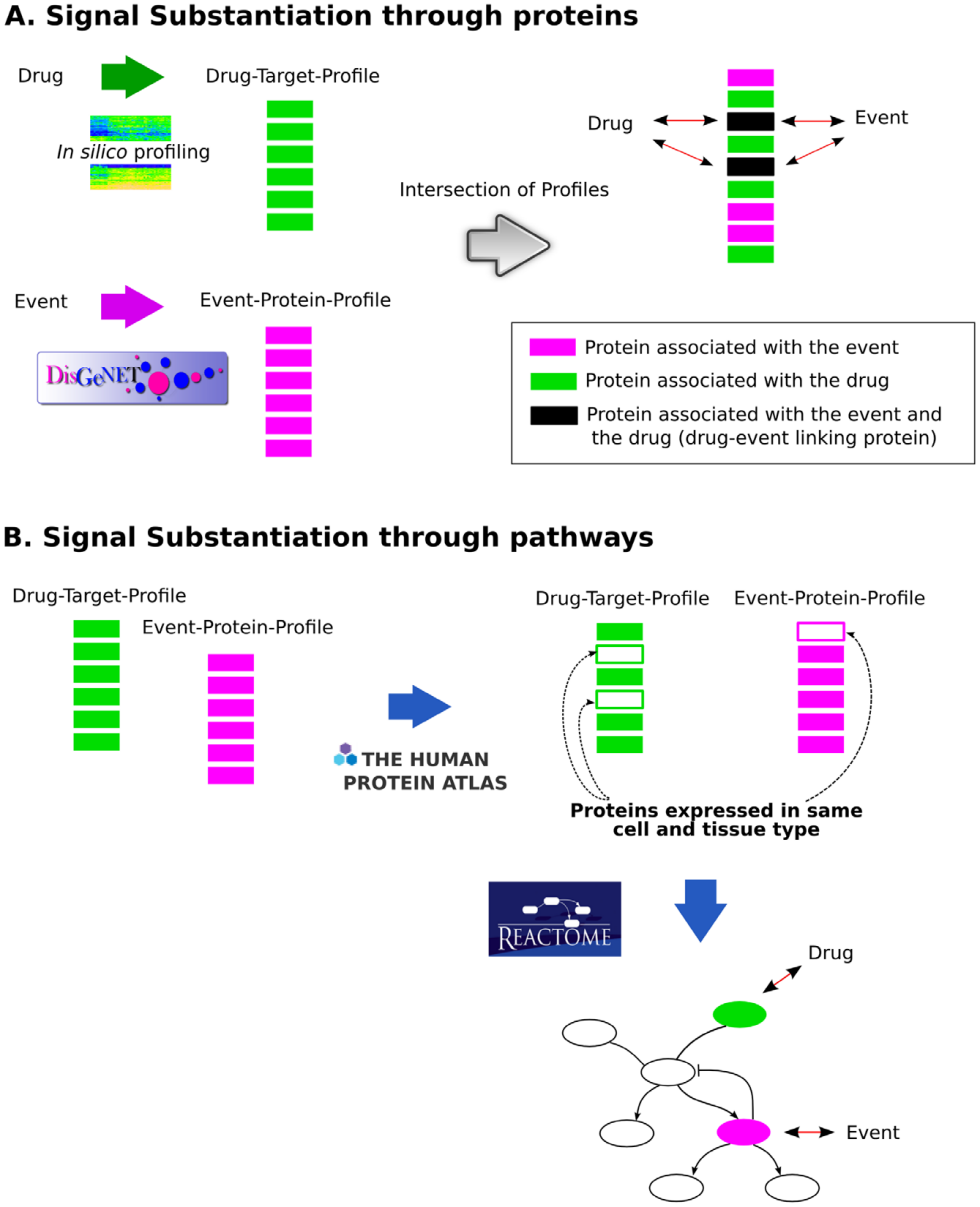
Schematic representation of the signal substantiation process. The signal substantiation process involves the automatic search for evidences that support the causal inference of the potential signal. A. Signal substantiation through proteins. The profile of targets of the drug and its metabolites is obtained by *in silico* profiling methods (Drug-Target-Profile). The profile of proteins associated with the clinical event is obtained by mining DisGeNET (Event-Protein Profile). The profiles are compared to find proteins in common in both profiles (Drug-Event Linking Proteins). The evidences that support the association of the drug and event with the Drug-Event Linking proteins are explored to determine if they support the causal inference of the signal. B. Signal substantiation through pathways. Proteins in the Drug-Target-Profile and in the Event-Protein Profile are searched in The Human Protein Atlas database to determine if they are expressed in the same tissue and cell type. Proteins that share expression at both levels (tissue and cell type) are used to query Reactome database, and pathways that contain at least one protein from the Drug-Target-Profile and one protein from the Event-Protein Profile are retrieved. Then, these pathways are explored to determine if they support the causal inference of the signal.

The here presented framework for the filtering and substantiation of drug safety signals consists of placing the potential signal in the context of current knowledge of biological mechanisms that might explain it. Essentially, we are searching for evidence that supports causal inference of the signal, i.e. feasible paths that connect the drug with the clinical event of the adverse reaction. The signal filtering analysis looks for evidence reporting the drug-event association in the biomedical literature and biomedical databases. The signal substantiation process considers two scenarios able to provide a causal inference of the signal (see Figure 1). First, we look for connections between the drug and the event through their associated protein profiles. Here, a connection is established if there are proteins in common between the drug-target and the event-protein profile (Figure 1A). Many ADRs are caused by altered drug metabolism for which genetic variants in metabolizing enzymes are often responsible. Consequently, we also consider drug metabolism phenomena as an underlying mechanism of the observed ADR by assessing if the drug metabolites are targeting proteins that are known to be associated with the clinical event. Second, the association between the drug and the clinical event can involve proteins that are not directly associated with the drug and the clinical event, but indirectly in the context of biological networks. The final consequence of the drug action is the observed clinical event. Thus, the proteins in the drug-target profile and event-protein profile are mapped onto biological pathways to evaluate if the drug and the event can be connected through biological pathways (Figure 1B).

Our approaches for *signal filtering* and *signal substantiation* were implemented using dedicated bioinformatics methods that are accessed through web services and integrated into processing pipelines by means of Taverna workflows. The substantiation workflow results can be visualized and analyzed by means of other bioinformatics tools such as Cytoscape [26], a software for network visualization and analysis. For the signal filtering process, we have implemented two Taverna workflows (ADR-FM and ADR-FD) that access data mined from databases such as DrugBank [27], DailyMed (http://dailymed.nlm.nih.gov/) and Medline®. A third Taverna workflow, (ADR-S), performs the signal substantiation process and was implemented by combining *in silico* target profiling, text mining and pathway analysis, among other bioinformatics approaches. More details about the implementation of web services and workflows can be found in the Methods section.

### Antipsychotic drugs and risk of cardiac arrhythmias

In the following section we describe the results of the analysis of potential drug safety signals as a proof of concept of the here proposed framework and tools.

In the 1990s, the occurrence of several cases of serious, life-threatening ventricular arrhythmias and sudden cardiac deaths, secondary to the use of non-cardiac drugs raised concerns with regulators [28]. In 1998, several drugs received a black-box warning in the US due to concerns regarding prolongation of the QT interval. Nowadays, it is known that many seemingly unrelated drugs can cause the prolongation of QT interval and Torsade de Pointes, which eventually may lead to fatal arrhythmias. For instance, cisapride, a drug for gastrointestinal protection, was withdrawn from the market in 2000 due to increased risk for QT prolongation. The first report of sudden cardiac death with an antipsychotic drug appeared in 1963 [29]. Since then, several studies found an increased risk for ventricular arrhythmias, cardiac arrest and sudden death associated with the use of antipsychotics [30], which can partly be explained by the prolongation of QT intervals observed with several antipsychotic drugs. It has been suggested that the mechanisms by which antipsychotics can cause prolongation of QT interval involve the potassium channel encoded by the KCNH2 gene that regulates myocyte action potential [31], [32]. Drugs blocking this potassium channel can slow down repolarization, which in turn may lead to the prolongation of the QT interval, eventually resulting in sudden cardiac death. We selected six antipsychotic drugs according to their risk of producing cardiac arrhythmias from [33] and from the QTdrugs database (http://www.qtdrugs.org) (Tables 1 and 2) and analyzed their association with the prolongation of the QT interval as defined in the EU-ADR project (referred to as QTPROL) using our signal filtering and substantiation workflows. The results of the filtering analysis (shown in Table 1) indicate that all drug-event associations are discussed in the literature or recorded in specialized databases, with the only exception of DrugBank that does not contain any information on the association of the selected drugs with QTPROL. When comparing both Medline-based filtering workflows, ADR-FM/MeSH and ADR-FD/Medline, the latter appears to be more sensitive as the number of abstracts found is generally higher (compare columns ADR-FM/MeSH and ADR-FD/Medline in Table 1). This difference might be explained by the different methods used by the two approaches. The MeSH®-based approach uses the MeSH terms assigned to each citation and the ADR-FD approach uses Natural Language Processing on title and abstracts to identify drug-event associations. Both Medline-based approaches can be compared with a PubMed query (“(QT or QTc) prolongation <one of the six antipsychotic drugs>”), which resulted in 2–3 times more abstracts being returned than by ADR-FD/Medline. This does not come as a surprise since PubMed searches for keyword co-occurrences at the abstract level. The workflows are more specific since they search at the sentence level (ADR-FD/Medline) or use additional information provided by the MeSH subheadings and the use of the pharmacological action (ADR-FM/MeSH). It should be noted that Medline is only one source of information to filter known signals; DrugBank and DailyMed are other, potentially complementary, sources. In the case of pimozide, no results are obtained from DailyMed®, since QT prolongation is not mentioned in the adverse reactions section but in the contraindications and warnings sections.

**Table 1:** Antipsychotics with low and high risk of producing prolongation of the QT interval (QTPROL) analyzed with the filtering workflows (ADR-FM and ADR-FD).

			Workflow			
			ADR-FM	ADR-FD		
Risk of QTPROL	Drug Name	ATC code	MesH	Medline	DailyMed	DrugBank
**Low**	Sulpiride	N05AL01	7	6	NA	0
	Quetiapine	N05AH04	7	18	2	0
	Olanzapine	N05AH03	14	20	1	0
**High**	Ziprasidone	N05AE04	15	38	3	0
	Pimozide	N05AG02	0	16	0	0
	Haloperidol	N05AD01	23	55	12	0

For the ADR-FD, the individual results obtained from the three different sources used (Medline, DailyMed and DrugBank) are shown. The table shows the number of records found in each case. NA: Not Available.

**Table 2:** Antipsychotics with low and high risk of producing prolongation of the QT interval (QTPROL) and the results of the substantiation process.

Risk of QTPROL	Drug Name	ATC code	Events	Drug-event linking proteins	p-value
**Low**	Sulpiride	N05AL01	None	None	None
	Quetiapine	N05AH04	LONG QT SYNDROME 1/2, 2, 2/5 and 2/3, TIMOTHY SYNDROME, Torsades de Pointes, Romano-Ward Syndrome	HERG (KCNH2, pKi 5.24)	0.0190
	Olanzapine	N05AH03	LONG QT SYNDROME 1/2, 2, 2/5 and 2/3, TIMOTHY SYNDROME, Torsades de Pointes, Romano-Ward Syndrome	HERG (KCNH2, pKi 4.64, pIC50 6.18)	0.0190
**High**	Ziprasidone	N05AE04	LONG QT SYNDROME 1/2, 2, 2/5 and 2/3, TIMOTHY SYNDROME, Torsades de Pointes, Romano-Ward Syndrome	HERG (KCNH2, pKi 6.77, pIC50 6.36)	0.1979
	Pimozide	N05AG02	LONG QT SYNDROME 1/2, 2/3, 2 and 2/5, TIMOTHY SYNDROME, Torsades de Pointes, Romano-Ward Syndrome, cardiac arrhythmia	HERG (KCNH2, pKi 6.99, pIC50 6.73), Cav1.2 (CACNA1C, pKi 6.7), hEAG1 (KCNH1, pIC50 6.2)	0.0025
	Haloperidol	N05AD01	LONG QT SYNDROME 2/3, 2, 2/5 and 1/2, TIMOTHY SYNDROME, Torsades de Pointes, Romano-Ward Syndrome	HERG (KCNH2, pKi 6.99, pIC50 6.73), Cav1.2 (CACNA1C, pKi 6.7), hEAG1 (KCNH1, pIC50 6.2)	0.0025

The columns display the risk of producing QTPROL for each drug, the drug name, the ATC code of the drug, the proteins that explain the connection between the drug and the event (Drug-event linking proteins), the clinical events associated with these proteins (Events), as well as p-values. For the drug-event linking proteins, the common protein name is given, and the Gene Symbol and the drug activity values of each drug-event linking protein (pKi or pIC50, average of the multiple values from different sources) are shown in parenthesis.

**Figure 2:**
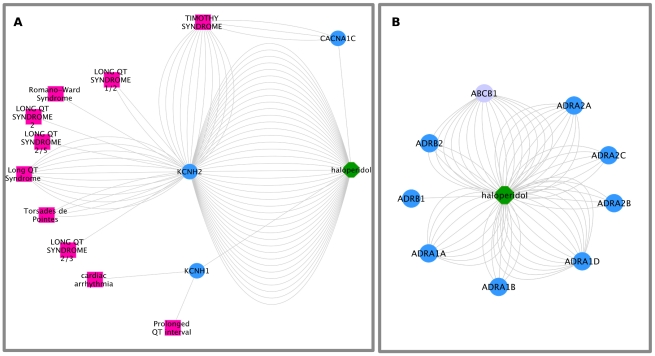
Cytoscape graph for QTPROL-haloperidol. The results of the ADR-S workflow can be visualized as a graph in which the nodes are proteins, compounds and clinical events. A: Detail of the network depicting the haloperidol targets, the proteins associated with QTPROL and the connection between them. The proteins encoded by the genes KCNH1, KCNH2 and CACNA1C constitute Drug-Event linking proteins between haloperidol and the terms corresponding to QTPROL. B: Detail of the targets of haloperidol, showing the adrenergic receptors (light blue) and the drug transporter encoded by the gene ABCB1 (purple). In both graphs, the multiple edges between two nodes represent different evidences for the corresponding association between the nodes.

We furthermore explored the mechanisms underlying the association between QTPROL and the selected antipsychotics using the substantiation workflow. The results are summarized in Table 2 (see Table 3 for a quick reference guide to gene and protein names discussed throughout the example) and Figure 2, which shows a detail of the Cytoscape graph representing the drug-protein-event network resulting from analyzing haloperidol and its association with QTPROL. For all the antipsychotic drugs, with the exception of sulpiride, connections are established through proteins associated with both, drug and event. All the connections between the drug and the event include the protein HERG encoded by the KCNH2 gene. All of the found connections are statistically significant except for ziprasidone (see Table 3). The high-risk antipsychotics haloperidol, ziprasidone and pimozide are potent potassium channel blockers (IC50 or K_i_ in the 0.1 µM range, Table 2). In the case of ziprasidone, it is worth to mention that one of the metabolites of the drug is predicted to bind to the protein HERG. Contrasting, olanzapine shows a lower activity on the protein HERG, while sulpiride has no activity on this protein. In addition to HERG, for the high-risk antipsychotics pimozide and haloperidol the drug and the event can be connected through the proteins encoded by the genes KCNH1 and CANCNA1C. In the case of KCNH1, which encodes the protein hEAG1, the ADR-S workflow provides evidence indicating that mutations in an animal model showed an association with prolonged QT interval and cardiac arrhythmia [34]. The mutations in the CACNA1C gene, which encodes the depolarizing long-lasting calcium current channel, are associated with Timothy syndrome, characterized by severe prolongation of the QT interval.

**Table 3:** List of proteins discussed in the text with their corresponding protein and gene identifiers.

Gene Symbol	Approved name (HGCN)	Other names	UniProt Accession	UniProt Identifier	NCBI Entrez Gene
**KCNH1**	potassium voltage-gated channel, subfamily H (eag-related), member 1	hEAG1, Kv10.1, eag, eag1, h-eag	O95259	KCNH1_HUMAN	3756
**KCNH2**	potassium voltage-gated channel, subfamily H (eag-related), member 2	HERG, Kv11.1, erg1	Q12809	KCNH2_HUMAN	3757
**CACNA1C**	calcium channel, voltage-dependent, L type, alpha 1C subunit	Cav1.2, CACH2, CACN2, TS	Q13936	CAC1C_HUMAN	775
**ABCB1**	ATP-binding cassette, sub-family B (MDR/TAP), member 1	Multidrug resistance protein 1, ABC20, CD243, GP170, P-gp	P08183	MDR1_HUMAN	5243

HGNC: HUGO Gene Nomenclature Committee (http://www.genenames.org/).

Interestingly, our analysis also indicates that the antipsychotics in our study have an important activity on adrenergic receptors (Figure 2 B).

Moreover, haloperidol shows activity on the drug transporter encoded by the gene ABCB1 (K_i_ 0.2 µM, Figure 1B). Similar activities are found for pimozide, whereas ziprasidone, olanzapine, sulpiride and quetiapine do not show activity on the transporter.

Regarding the substantiation through pathways, for haloperidol and pimozide we found several Reactome pathways (Integration of energy metabolism, Axon guidance, Synaptic transmission, Signaling by GPCRs and Diabetes pathways), which connect the drug and the event, and where the involved proteins are expressed in cardiac tissues. It is likely that the effect of a drug on its target proteins will affect proteins in their direct neighborhood in the biological pathway. Hence, we computed the average shortest path length between pairs of drug and event associated proteins in the Reactome pathways and compared them to the average shortest path length between randomly selected drug and event proteins. Interestingly, for all five antipsychotic drugs, the drug and event proteins are in close proximity in the Reactome pathways with average shortest path lengths between 2 and 3, which are significantly shorter than the average shortest path length of 5 of randomly selected drug and event proteins (p-value< = 0.05).

In summary, the ADR-S workflow provides different hypotheses explaining the antipsychotics-induced QTPROL, including the direct action of the drug on proteins associated with the clinical event (e.g. HERG), the cross-talk between different biological processes (adrenergic signaling and cardiac action potential), and the differential distribution of drugs among tissues (due to inhibition of transporters exerted by the drug). Moreover, it also highlights several interesting evidences that might explain the differences between low and high-risk antipsychotics.

### Analysis of drug-event pairs from SIDER

In addition to the example case presented above, the ADR-S workflow was evaluated on a large-scale data set. The SIDER database was used to extract drug-event pairs (see Methods). Here, an event refers to a known side effect of a drug compiled from package inserts of the drugs from several public sources [25]. For a total of 28251 drug-event pairs, 6108 (4265 with p-value< = 0.01) pairs can be directly linked through at least one protein connecting the drug with the side effect. Interestingly, 2692 (44%) of the 60108 drug-event pairs are connected by means of the drug metabolites. Moreover, the substantiation through pathways module finds connections between 21526 pairs (10789 with p-value< = 0.01). This quantitative analysis should be followed by a thorough qualitative study on selected drug-event pairs of interest in order to explore the found connections and derive mechanistic hypothesis. Hence, we make the results of the analysis available as Supplementary Material (Dataset S1 and S2).

## Discussion

Recent studies highlight the use of disparate data sets in the study of ADRs, enabled by bioinformatics methodologies. Combining the study of protein–drug interactions on a structural proteome-wide scale with protein functional site similarity search, small molecule screening, and protein–ligand binding affinity profile analysis, Xie and colleagues [35] have elucidated a possible molecular mechanism for the previously observed, but molecularly uncharacterized, side effect of selective estrogen receptor modulators (SERMs). In another study, the side effect information from prescription drug labels was exploited to identify novel molecular activities of existing drugs [25]. The Unified Medical Language System (UMLS) Metathesaurus® [36] was used as a vocabulary for the side effects, and a weighting scheme to account for the rareness and interdependence of side effects was developed. Since similarity in side effects correlated with shared targets between drugs, side effect similarity was used to predict novel targets between any two “unexpected” drug pair [25]. In another study, Berger and colleagues used a computational systems biology approach to analyze drug-induced long QT syndrome, and showed that the analysis of a human protein interaction network associated with congenital long QT syndrome can be used to predict new gene variants for long QT syndrome, to explain the complexity of the adverse drug reaction, and to predict the susceptibility of new drugs to cause long QT syndrome [32].

All these examples illustrate how computational approaches are paving the way toward elucidating the molecular mechanisms of ADRs. The here presented framework follows this direction, by traversing and integrating information from the chemical domain, through genes and proteins, molecular and cellular networks, and finally to the clinical domain. The filtering workflows interrogate specialized databases and literature repositories in order to determine the novelty of a drug-event association. On the other hand, the substantiation framework seeks to find hypotheses that might explain drug-induced clinical events by looking for evidences supporting causative connections between the drug, its targets, and their direct or indirect (through biological pathways) association to the clinical event. The signal substantiation process can be framed as a closed knowledge discovery process, analogous to the Swanson model based on hidden literature relationships [37], which we extend by considering not only relationships found in the literature, but also relationships discovered by mining other data sources or found by applying different bioinformatics methods (*vide infra*). For a drug-event association, we collect information about the drug-targets by querying publicly available databases and by applying *in silico* drug-target profiling methods [38]. In parallel, we retrieve information about the genes and proteins associated with the clinical event from a database covering knowledge about the genetic basis of diseases [39]. Then, we combine these two pieces of information under the following assumption: if the disease phenotype elicited by the drug is similar to the phenotype observed in a genetic disease, then the drug acts on the same molecular processes that are altered in the disease. This can be regarded as *phenocopy*, a term originally coined by Goldschmidt in 1935 [40] to describe an individual whose phenotype, under a particular environmental condition, is identical to the one of another individual whose phenotype is determined by the genotype. In other words, in the phenocopy the environmental condition mimics the phenotype produced by a gene. In the case of ADRs, the environmental condition is represented by the exposure to the drug, whose effect mimics the phenotype (disease) produced by a gene in an individual. In this way, we can capitalize on all the knowledge about the genetic basis of diseases to explore mechanisms underlying ADRs.

We illustrate our approach by analyzing a clinically relevant drug safety signal: prolongation of the QT interval (QTPROL) leading to cardiac arrhythmias produced by a set of antipsychotic drugs. The results of the filtering workflows show that the association of QTPROL with the antipsychotic drugs has been extensively discussed in the literature and is documented in specialized databases. On the other hand, the substantiation workflow provides different hypotheses explaining the antipsychotics-induced QTPROL. First, we were able to confirm the widely accepted mechanism proposed for drug-induced QTPROL, in which the drug blocks the potassium channel HERG (encoded by the KCNH2 gene) and this blockade leads to a prolongation of the QT interval [41], [42]. The known association of congenital long QT syndrome being associated with mutations in the KCNH2 gene furthermore supports this concept [38], [39]. Interestingly, our analysis reveals that high-risk antipsychotics show higher activities on the potassium channel than low-risk antipsychotics (see Table 2), suggesting that the strength of binding might explain the different risks of observing the side effect for different antipsychotics. For all except one antipsychotic (ziprasidone), the associations between the drugs and QTPROL are statistically significant (p-value< = 0.01). We want to point out, that even for ziprasidone with a higher p-value, the evidences provided by the workflow give enough confidence to establish the hypothesis of the blockage of HERG being related with QTPROL. We believe that each drug-event pair and the evidences provided by the workflows have to be studied carefully in order to generate hypotheses valid to be tested. We furthermore find a connection of high-risk antipsychotics and QTPROL through other proteins different from HERG, suggesting that the prolongation of the QT interval might result from the effect of the drugs on other channel proteins regulating the action potential. In addition to the direct blockade of channels creating ion currents involved in the action potential, other factors can be considered for the mechanism of antipsychotics-induced QTPROL. Adrenergic activation due to stress can precipitate cardiac arrhythmias [35]; in fact, the main treatment for patients with congenital long QT syndrome is beta-adrenergic blocking [41]. Alpha and beta-receptors agonists produce an inhibition of the potassium channel leading to the prolongation of QT [34]. Interestingly, our results indicate that the antipsychotics in our study have an important activity on adrenergic receptors. Haloperidol has been reported to act as partial agonist in cerebral alpha-adrenergic receptors [43]. Hence, our results suggest that the modulation of adrenergic signaling by haloperidol might be an additional factor resulting in the inhibition of the potassium repolarizing current. Thus, in the case of haloperidol, direct inhibition by the drug combined with an indirect mechanism involving the activation of beta adrenergic signaling might lead to HERG blockade. These findings are in line with evidences supporting the notion that ADRs may often be caused by the combined action of multiple genes [9].

We furthermore found that activities of haloperidol and pimozide on the drug transporter encoded by the gene ABCB1 (K_i_ 0.2 µM, Figure 1B), while ziprasidone, olanzapine, sulpiride and quetiapine do not show activity on this transporter. Titier and colleagues studied the myocardium to plasma concentration ratio of several antipsychotic drugs, reporting ratios of 2.7 for olanzapine and 6.4 for haloperidol [43]. Therefore, the different distributions of the antipsychotics between plasma and the heart could be another factor influencing the varying risk of different antipsychotic drugs to induce QTPROL.

Regarding the analysis through biological pathways, our workflow does not provide novel hypotheses that might explain drug-induced QTPROL in addition to the above presented hypotheses. Nevertheless, it is interesting that the drug target proteins and event-associated proteins are closely located in the Reactome pathways. All in all, a detailed analysis of the generated paths might add valuable information about the mechanism underlying the drug adverse reaction. Ultimately, the usefulness of the pathway module strongly depends on the drug-safety signal of interest. For example, the cholesterol-lowering drug cerivastatin was withdrawn from the market in 2001 due to its fatal risk to induce rhabdomyolysis leading to kidney failure [44]. While the ADR-S workflow connects cerivastatin and rhabdomyolisis through proteins and pathways, it only finds a meaningful connection between the drug and acute renal failure through the pathway module. Hence, in this example the pathway module adds valuable information to the analysis. We also want to mention some limitations of the pathways module. The publicly available information on pathways is not complete, and the level of detail differs between the pathways. Moreover, the Reactome pathways used are at a very high level in the Reactome hierarchy and can be very general; hence the substantiation results need to be carefully analyzed in order to determine if the connection found between the drug and the event represents a plausible explanation of the ADR.

In summary, using antipsychotics and their risk to induce QTPROL, we showed that the filtering workflows are able to extract relevant information from the literature and dedicated databases. We also showed that the substantiation workflow provides different hypotheses explaining the antipsychotics-induced QTPROL. These hypotheses include the direct action of the drug on proteins associated with the clinical event (e.g. HERG), the cross-talk between different biological processes (adrenergic signaling and cardiac action potential), and the differential distribution of drugs among tissues (due to inhibition of transporters exerted by the drug). Moreover, the analysis also highlights several interesting evidences that might explain the differences between low and high-risk antipsychotics. In addition, we provide the results of a large-scale analysis of drug-side effect pairs from SIDER and show that about 22% of the known side effects of drugs might involve direct effects of drugs on proteins being associated with the events. This relatively small number is not surprising because not all drug side effects can be attributed to the direct action of the drug onto its targets, such as on-target and off-target pharmacological effects. Other mechanisms of drug toxicity have been discussed. For example, metabolites can react with nucleophiles including DNA, which can trigger regulatory processes leading to inflammation, apoptosis and necrosis [45]. Moreover, the workflow uses public data sources on drug-target and event-protein associations, which are not complete. Interestingly, almost half (44%) of the direct connections through proteins involve metabolites of the drugs. This finding is in good agreement with current opinion on the relevance of drug metabolism for drug adverse reactions [9]. The pathway module connects many more drug-side effect pairs. Although, the results of our workflow for each drug-side effect pair have to be carefully analyzed in detail, this finding suggests that the indirect connection of drug and event in the context of biological networks plays an important role. We want to stress that the substantiation workflow provides a variety of evidences, such as the binding strength of the drug to its targets, as well as the provided literature sources supporting the associations of proteins to the events. All pieces of evidence need to be carefully considered to generate hypotheses of mechanisms that are valid to be further tested.

Both filtering and substantiation workflows are available to the community and allow a systematic and automatic analysis of drug safety signals detected by mining clinical records, providing a user-friendly framework for the analysis of drug-event combinations. We believe that with the availability of such tools for *in silico* experimentation, research on the mechanism that underlies drug-induced adverse reactions will be facilitated, which will have great impact in the development of safer drugs.

## Methods

The signal filtering and substantiation framework has been implemented by means of software modules that perform specific tasks of the processes. To allow access and integration of the modules in high-level analysis pipelines, the modules were implemented as web services and combined into data processing workflows to achieve the aforementioned signal filtering and signal substantiation. To standardize data exchanges between the different web services, we have developed two complementary schemas using XSD to define a common XML interoperability structure. The first one describes general data types (http://bioinformatics.ua.pt/euadr/common_types.xsd) and the second one defines the specific types needed for signal filtering and substantiation in the context of the EU-ADR project (http://bioinformatics.ua.pt/euadr/euadr_types.xsd). Both schemas allow a smooth integration of the different modules in Taverna workflows, by enabling content and structure validation for the workflow input and output XML files. Moreover, the use of schemas facilitates further data transformations, for example, by applying XSL transformation to XML files of the signal substantiation workflow to create XGMML file graphs that can be visualized with Cytoscape. The workflows and web services are described in the following sections. All workflows have been implemented and tested using Taverna Workflow Management system version 2.2.

### Workflows: Signal filtering

We have implemented two workflows for signal filtering. The ADR-FM workflow is a MeSH®-based approach to find drug-event pairs in Medline® citations. The ADR-FD workflow uses text-mining to find the drug-event pairs in Medline® abstracts, databases such as DrugBank and drug labels available at DailyMed®.

#### ADR-FM

The aim of this signal filtering workflow is to automate the search of publications related to a given drug-adverse event association. It is based on an approach that uses the MeSH® annotations of Medline® citations, in particular the subheadings “chemically induced”, “adverse effects” and “Pharmacological Action” [46]. This workflow offers the opportunity to automatically determine if an ADR has already been described in Medline®. However, the causality relationship between the drug and an event may be judged only by an expert reading the full text article and determining if the methodology of this article was correct and if the association is statically significant, among other factors. The workflow uses the method *getListPublis* of the UB2_EUADR web service (Table 4).

**Table 4:** Availability of web services and workflows.

URL	Description	Type
http://bioinformatics.ua.pt/euadr/common_types.xsd	XSD schema defining common data types.	XSD schema
http://bioinformatics.ua.pt/euadr/euadr_types.xsd	XSD schema defining specific types used in the EU-ADR project.	XSD schema
http://lesim.isped.u-bordeaux2.fr/axis2/services/UB2_EUADR?wsdl	Web service with the method getListPublis	Web service endpoint
http://aneurist.erasmusmc.nl/euadr-manager-db/euadr-service-db?wsdl	Web service with the method get FilteredRelations	Web service endpoint
http://cgl.imim.es/axis2/services/cglAlertService?wsdl	Web service with the methods getSmileFromATC and getUniprotListFromSmile	Web service endpoint
http://ibi.imim.es/axis2/services/AdrPathService?wsdl	Web service with the methods getDiseaseAssociatedProteins andgetPathways	Web service endpoint
http://www.myexperiment.org/workflows/2280.html	ADR-FM workflow	Workflow
http://www.myexperiment.org/workflows/2279.html	ADR-FD workflow	Workflow
http://www.myexperiment.org/workflows/1988.html	ADR-S workflow	Workflow

#### Workflow input

The ADR-FM workflow accepts two inputs, the ATC (Anatomical Therapeutic Chemical, http://www.whocc.no/atc_ddd_index/) code of the drug at the 7 digits level (e.g. M01AH02 for rofecoxib) and the event represented by a string as defined in the EU-ADR project (see Table 5).

**Table 5:** Event codes and names of events as defined in the EU-ADR project [48], [49].

Event code	Event name
BE	Bullous Eruptions
AS	Anaphylactic Shock
ARF	Acute Renal Failure
AMI	Acute Myocardial Infarction
ALI	Acute Liver Injury
CARDFIB	Cardiac Valve Fibrosis
UGIB	Upper gastrointestinal bleeding
RHABD	Rhabdomyolysis
PANCYTOP	Aplastic anemia/Pancytopenia
NEUTROP	Neutropenia/Agranulocytosis
QTPROL	QT Prolongation

#### Workflow output

The workflow returns an XML file and an HTML page summarizing the results, showing the PubMed identifiers of the retrieved citations grouped by publication type. A chart of the number of retrieved citations per year is generated using Google Charts Tools (http://code.google.com/apis/chart/).

#### ADR-FD

This workflow looks for associations between drugs and side effects that have been recorded in literature (Medline®) or in databases (DailyMed® and Drugbank). These resources have been indexed, and co-occurrences of drugs (corresponding to ATC codes) and side effects as defined in the EU-ADR project were captured and stored in a database. Briefly, all abstracts in the Medline database were split into sentences, and all sentences were indexed by the concept-recognition tool Peregrine [47] to find drugs and adverse events. A chi-square test was performed to check if the probability of the drug and the adverse event co-occurring together in a sentence was significantly different than would be expected by chance. Regarding the databases, for each entry in DrugBank a field specifying ATC codes and a field listing potential adverse events were extracted and processed by Peregrine. DailyMed® contains Summary Product Characteristics (SPCs) of drugs. Each SPC was parsed to extract the “title” field (containing the drug name) and the “adverse reaction” and “boxed warning” fields (containing the adverse events). These fields were subsequently indexed by Peregrine and the output was processed to link ATC codes to UMLS concept identifiers of adverse events. The workflow uses the method *get FilteredRelations* (Table 4), which provides relationships between a drug and an event in one or more of the data sources.

#### Workflow input

The ADR-FD workflow accepts three inputs: the ATC code of the drug at the 7-digit level (e.g., M01AH01 for celecoxib), the event as defined in the EU-ADR project (Table 5), and the data resources in which the specified drug-event pair is sought (Medline®, DailyMed®, or DrugBank).

#### Workflow output

The output of the workflow consists of a list of links to entries in the input data sources (Medline® abstracts, DailyMed® SPCs, or Drugbank cards) in which the input drug-event association is mentioned. The output is generated in XML format and in HTML format.

### Workflows: Signal substantiation

#### ADR-S

The ADR substantiation (ADR-S) workflow seeks to establish a connection between the clinical event and the drug through (i) proteins targeted by the drug (or by its metabolites) and associated with the clinical event and (ii) biological pathways. In the first connecting path, the link between the drug and the event is established through the set of proteins in common between the Drug-Target-Profile and the Event-Protein-Profile (Figure 1A). In the second path, the link is established through a set of proteins that are part of the same biological pathway (Figure 1B). For example, consider a protein A targeted by the drug and a protein B associated with the clinical event, and both proteins A and B are part of the same biological pathway C. Then, the drug and the event are connected through biological pathway C (see more details in the description of the service *adrPathService*). Two SOAP web services (*cglService* and *adrPathService*) allowing access to databases and bioinformatics modules relevant for the signal substantiation have been implemented (Table 4). A tutorial describing how to use the ADR-S workflow can be found in the Supportive information (Protocol S1) and at http://ibi.imim.es/ADR_Substantiation.html.

#### getSmileFromATC (cglAlertService)

This method accepts as input a drug encoded by the ATC code at the 7-digits level and provides as output the chemical structure by means of SMILE (Simplified Molecular Input Line Entry Specification).

#### getUniprotListFromSmile (cglAlertService)

This method accepts as input a drug or metabolite encoded by a SMILE and returns a list of proteins that are related to the drug (Drug-Target-Profile). We use known drug-target associations (Table 6) and extend them with *in silico* target profiling methods [38]. Drug metabolites are obtained from a commercial database (GVK Biosciences) and are also processed by *in silico* target profiling. The evidences that support each drug-target relationship, such as the binding affinity of the compound to the protein or the source database, are provided.

**Table 6:** Drug-target databases used in the ADR-S workflow.

Database	Description	URL
AffinDB	The Affinity Database (AffinDB) contains affinity data for protein-ligand complexes of the PDB.	http://pc1664.pharmazie.uni-marburg.de/affinity/
BindingDB	BindingDB is a public, web-accessible database of measured binding affinities for biomolecules, genetically or chemically modified biomolecules, and synthetic compounds.	http://www.bindingdb.org/bind/index.jsp
ChemblDB	ChEMBL is a database of bioactive drug-like small molecules, it contains 2-D structures, calculated properties (e.g. logP, Molecular Weight, Lipinski Parameters, etc.) and abstracted bioactivities (e.g. binding constants, pharmacology and ADMET data).	https://www.ebi.ac.uk/chembl/
DrugBank	DrugBank is a unique bioinformatics and chemoinformatics resource that combines detailed drug (i.e. chemical, pharmacological and pharmaceutical) data with comprehensive drug target (i.e. sequence, structure, and pathway) information.	http://www.drugbank.ca/
hGPCRlig	hGPCRlig is a bank of 3-D human G-Protein Coupled Receptor models and their known ligands.	http://cheminfo.u-strasbg.fr:8080/hGPCRlig
IUPHARdb	IUPHARdb incorporates detailed pharmacological, functional and pathophysiological information on G Protein-Coupled Receptors, Voltage-Gated Ion Channels, Ligand-Gated Ion Channels and Nuclear Hormone Receptors.	http://www.iuphar-db.org/index.jsp
MOAD	Binding MOAD's goal is to be the largest collection of well resolved protein crystal structures with clearly identified biologically relevant ligands annotated with experimentally determined binding data extracted from literature.	http://www.bindingmoad.org/
NRacl	NRacl is an annotated compound library directed to nuclear receptors as a means for integrating the chemical and biological data being generated within this family. All data incorporated in NRacl were collected from public sources of information, mainly reviews and medicinal chemistry journals of the last 10 years [53].	[53]
PDSP	This service provides screening of novel psychoactive compounds for pharmacological and functional activity at cloned human or rodent CNS receptors, channels, and transporters.	http://pdsp.med.unc.edu/indexR.html
PubChem	PubChem provides information on the biological activities of small molecules. It is a component of NIH's Molecular Libraries Roadmap Initiative.	http://pubchem.ncbi.nlm.nih.gov/

#### getDiseaseAssociatedProteins (adrPathService)

This method accepts as input a clinical event (encoded as a list of UMLS® concept identifiers or as a string as defined in Table 5) and returns a list of proteins associated to the event (Event-Protein-Profile), by interrogating the DisGeNET database [39]. Evidences that support each association, including the association type, source database, publications discussing the association, and in the case of text-mining derived associations, the sentence that reports the gene-disease association, are provided.

#### getPathways (adrPathService)

This method assesses if proteins associated with the drug and the event are annotated to the same biological pathway by interrogating Reactome [48]. In general, pathway databases such as Reactome contain a canonical, general description of biological processes and pathways [49]. These pathways can be found in different cell types and tissues, or in different time points in the life of an organism; however, not all the pathway components might be active in all circumstances. Combining information from pathways with protein expression in tissues and cell types can result in a cell and tissue type specific view of a given pathway. Thus, this method combines annotation of proteins to pathways with information of protein expression in cells and tissues. Briefly, we determine if the proteins associated with the drug and the event are expressed in the same tissue and cell type according to the The Human Protein Atlas version 7.1 [50]. Only the proteins that share expression at both levels (tissue and cell type) are kept for the next step. Then, for this list of proteins, we retrieve all annotations to pathways using the Reactome web service (Figure 1B). The input of the method is composed of two lists of UniProt identifiers and the output is an XML document listing the pathways, the annotated proteins and their expression profile.

#### Workflow input

The substantiation workflow has five input ports, called *atc*, *event*, *eventType*, *eventName*, and *cytoscape*. The signal is represented by the ATC code of the drug at the 7-digits level (e.g. M01AH02 for celecoxib) and the event, which is defined by the three input ports *event*, *eventName* and *eventType*. We allow two different types of event definitions: events as defined in the EU-ADR project (Table 5), and events defined by a set of UMLS® concept identifiers. The input port *eventType* is then used to distinguish between the two definitions for events. The *eventName* can be set by the user and is only required for user-friendly visualization of the results. The *cytoscape* input port defines the location of the local Cytoscape installation (e.g. /home/user/cytoscape-v2.7.0); it is optional and only required for the visualization of the signal substantiation results (Figure 2).

#### Workflow output

The output of the signal substantiation workflow consists of 7 ports representing different layers of the results. Besides the raw outputs from the individual web services (*drugTargetOutput* and *diseaseProteinOutput*), the protein profile of the drug or its metabolites (*drugTargets*), and the protein profile of the event (*diseaseProteins*) are provided. The signal substantiation workflow combines two ways of connecting drug and event, through proteins or through biological pathways. The outcome of these results is shown to the user during workflow execution by pop-up windows. The list of connecting proteins, that is, the protein annotated to both the drug and the event is provided (*connectingProteins*). For a user-friendly visualization and analysis of the results, a Cytoscape graph (*CytoscapeResultGraph*) is generated. The graph is composed of three types of nodes: drug, event, and proteins, and two types of edges: drug-protein, protein-event. The attributes of the edges contain supporting information for each association, such as source databases, association type, binding value for the drug, etc. (Tables 7 and 8). As result of the pathway analysis the output port *connectingPathways* provides a list of all pathways connecting drug and event that can be visualized as HTML file.

**Table 7:** Node attributes in the Cytoscape graph.

Entity	ID	SMILE	styleName	nodeType
**Drug**	Internal identifier for the node in the network.	The SMILE string corresponding to the drug structure.	Common name for the node.	Drug
	The ATC code for the drug.		The generic drug name.	
**Metabolite**	Internal identifier for the node in the network.	Not provided	Common name for the node.	Drug
	Internal identifier for the metabolite.		Numbered metabolite.	
**Event**	Internal identifier for the node in the network.	Not applicable	Common name for the node.	Event
	The UMLS® CUI for the event.		Name of the UMLS® CUI concept extracted from UMLS®.	
**Protein**	Internal identifier for the node in the network.	Not applicable	Common name for the node	Protein
	The UniProt accession number for the protein.		Gene symbol for the protein as in UniProt.	

**Table 8:** Edge attributes in the Cytoscape result graph.

	ID	bindingValue	evidenceLink	evidenceSource	evidenceType	relationshipType
**Drug-protein**	Internal identifier constructed of the ATC code of the drug and the UniProt identifier of the protein.	The binding affinity value as reported in the original database.	Not applicable	Database providing the association.	OBSERVATIONAL for associations taken from databases. SIMILARITY for associations from *in silico* profiling.	BINDS for drug-target binding
**Metabolite-protein**	Internal identifier constructed of the metabolite identifier and the UniProt identifier for the protein.	The binding affinity value as reported in the original database or transferred during *in silico* profiling.	Not applicable	Database providing the association.	OBSERVATIONAL for associations taken from databases. SIMILARITY for associations from *in silico* profiling.	BINDS for metabolite-target binding.
**Event-protein**	Internal identifier constructed of the UMLS® CUI concept and the UniProt identifier of the protein.	Not applicable	PubMed identifier of the publication supporting the association, empty if not available.	Database providing the association.	OBSERVATIONAL for associations from curated databases. TEXT-MINING for text-mining derived associations.	Association type according to the gene-disease association ontology available in [23].

#### Workflow run

The different web services run in parallel. The drug ATC code is first processed by the module getSmileFromATC, which returns the SMILE code of the drug. The SMILE code is then further processed by the module getUniprotListFromSmile, which returns the relationships between the drug and its targets, including targets of the metabolites of the drug. The event is processed by the module getDiseaseAssociatedProteins, which returns relationships between the event and associated proteins. The lists of proteins associated with drug or event are extracted by means of Java scripts using XPath queries and are further processed to remove duplicates. The module ConvertToCytoscapeGraph converts the output of the web services to a Cytoscape graph for user-friendly visualization by means of XSL transformation. For the signal substantiation through proteins, the two protein profiles are combined to determine the proteins in common between the two profiles (module CheckIntersection). For the signal substantiation through pathways, the two protein profiles are subjected to the module getPathways, which returns a list of pathways to which at least one drug and one event protein that are expressed in the same tissue are annotated to. The output is further processed by module ConvertToHTML, which generates an HTML file listing the pathways that connect the drug and the event.

### Analysis of drug-side effects from SIDER

A dataset of drug-side effects was downloaded from SIDER (December 2011) [25]. We restricted the SIDER dataset of total 61102 drug-event associations to 28251 associations between 492 drugs and 974 side effects by (i) mapping the used drug and event identifiers to the vocabularies used in our framework (ATC codes for drugs and UMLS concept identifiers for adverse events), and (ii) restricting to drugs and events for which protein annotations were available. P-values were computed using Fisher exact test and FDR was used to correct for multiple hypothesis testing.

### Shortest path analysis

We used the protein-protein interaction representation of the Reactome pathways (http://www.reactome.org/download/current/homo_sapiens.interactions.txt.gz, January 2012) to calculate the shortest path between any pair of antipsychotic drug and QTPROL associated proteins. For this purpose, we used the implementation of the Dijkstra algorithm in the Perl package Graph (http://search.cpan.org/~jhi/Graph-0.94/lib/Graph.pod). We then computed the average shortest path length for randomly chosen combinations of drug and event proteins and used a one-sided t-test to assess if the shortest path between the drug and event proteins as observed in our analysis was shorter than compared to random.

### Event definition and terminology mapping

The EU-ADR project focuses on a selection of adverse drug reactions that are monitored in electronic health records and further analyzed by the filtering and substantiation workflows [7], [8]. These events were defined in terms of UMLS Metathesaurus® concept identifiers as described in [51], [52]. The event codes and names as defined in the EU-ADR project are listed in Table 5. The mapping of events codes or strings to UMLS Metathesaurus® concept identifiers and other vocabularies such MeSH® and OMIM is implemented within the web services. The ADR-S workflow accepts events as defined in the EU-ADR project or any other clinical event defined by UMLS concept identifier. The UMLS concept identifiers are processed to map them to MeSH® and OMIM identifiers using the UMLS Metathesaurus®.

### Availability

The availability of web services and workflows presented in this work is detailed in Table 4.

## Supporting Information

Dataset S1 Results of the large-scale analysis of drug-side effects from SIDER using the module ADR-S through proteins.
(TXT)

Dataset S2 Results of the large-scale analysis of drug-side effects from SIDER using the module ADR-S through pathways.
(TXT)

Protocol S1 Tutorial for the ADR-S workflow.
(PDF)
