# Correction: Automatic Filtering and Substantiation of Drug Safety Signals

**DOI:** 10.1371/annotation/695450aa-95a0-491d-804d-470cbfa861e8

**Published:** 2012-05-04

**Authors:** Anna Bauer-Mehren, Erik M. van Mullingen, Paul Avillach, María del Carmen Carrascosa, Ricard Garcia-Serna, Janet Piñero, Bharat Singh, Pedro Lopes, José L. Oliveira, Gayo Diallo, Ernst Ahlberg Helgee, Scott Boyer, Jordi Mestres, Ferran Sanz, Jan A. Kors, Laura I. Furlong

Throughout the article the citation for reference [25] was incorrect. The correct citation is "Kuhn M, Campillos M, Letunic I, Jensen LJ, Bork P (2010) A side effect resource to capture phenotypic effects of drugs. Molecular Systems Biology 6:343."

