# Supplementary material for: Automatic Filtering and Substantiation of Drug Safety Signals
Source: PLoS Comput Biol. 2012 Apr 5;8(4):e1002457. doi: 10.1371/journal.pcbi.1002457 (PMC3320573; doi:10.1371/journal.pcbi.1002457)
Supplement: Protocol S1 — Tutorial for the ADR-S workflow. (PDF) [file pcbi.1002457.s003.pdf]

ADR substantiation workflow tutorial

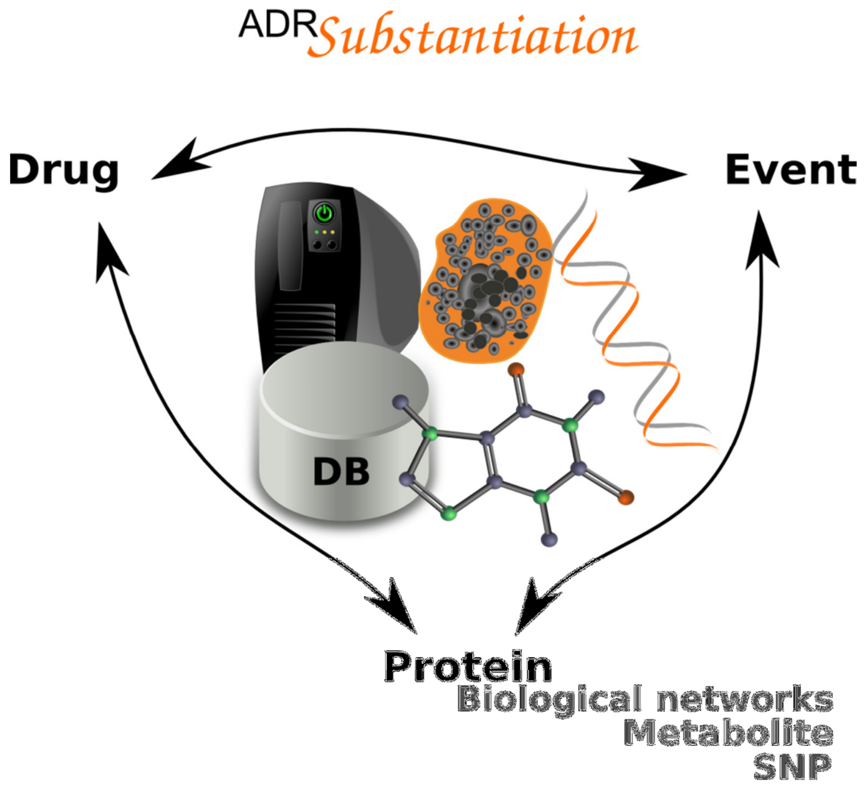

Contents

1. Summary ... 2
2. Description of the workflow ... 3
The substantiation concept ... 3
Implementation of the substantiation concept ... 4
3. License ... 7
4. Requirements ... 7
a. Install Taverna ... 8
b. Install Cytoscape ... 8
5. How to run the workflow ... 8
a. Launch Taverna ... 8
b. Open the workflow ... 8
c. Run the workflow ... 9
d. Workflow results ... 14
Cytoscape graph results ... 14
Pathway results ... 17
e. Invalid Input values ... 17
6. EU-ADR events ... 17
7. Service ports ... 18
8. Workflow URL ... 18
9. References ... 18
10. Tables ... 19
11. Funding ... 21
12. Information about this document ... 21

1. Summary

Drug safety issues pose serious health threats to the population and constitute a major cause of mortality worldwide. Due to the prominent implications to both public health and the pharmaceutical industry, it is of great importance to unravel the molecular mechanisms by which an adverse drug reaction can be potentially elicited. These mechanisms can be investigated by placing the pharmaco-epidemiologically detected adverse drug reaction in an information-rich context and by exploiting all currently available biomedical knowledge to substantiate it. We present a computational framework for

the biological annotation of potential adverse drug reactions. The proposed framework seeks to provide a biological explanation (*signal substantiation*) by exploring mechanistic connections that might explain why a drug produces a specific adverse reaction. The mechanistic connections include the activity of the drug, related compounds and drug metabolites on protein targets, the association of protein targets to clinical events, and the annotation of proteins (both protein targets and proteins associated with clinical events) to biological pathways. Hence, the substantiation workflow (ADR-S workflow) integrates modules for *in silico* drug-target profiling, and analyses based on gene-disease networks and biological pathways. The ADR-S workflow offers a novel approach to explore the molecular mechanisms underlying adverse drug reactions.

This tutorial accompanies the article:
Bauer-Mehren A, van Mullingen EM, Avillach P, Carrascosa MC, Singh B, Garcia-Serna R, Lopes P, Oliveira JL, Diallo G, Mestres J, Ahlberg Helgee E, Boyer S, Sanz F, Kors JA, Furlong LI. **Automatic filtering and substantiation of drug safety signals**, *submitted*

2. Description of the workflow

The substantiation concept

The substantiation concept for drug safety signals here presented consists of placing the signal in the context of current knowledge of biological mechanisms that might explain it. Essentially, we are searching for evidence that supports causal inference of the signal, i.e. feasible paths that connect the drug with the clinical event of the adverse reaction. The signal substantiation process can be framed as a closed knowledge discovery process, analogous to the Swanson model based on hidden literature relationships [1]. We extend this framework by considering not only relationships found in the literature, but also relationships discovered by mining other data sources or found by applying different bioinformatics methods (*vide infra*). For a drug-event association, we collect information about the targets of the drug by querying publicly available databases and by applying drug-target profiling methods [2]. In parallel, we retrieve information about the genes and proteins associated with the clinical event from a database covering knowledge about the genetic basis of diseases [3]. Then, we combine these two pieces of information under the following assumption: if the disease phenotype elicited by the drug is similar to the phenotype observed in a genetic disease, then the drug acts on the same molecular processes that are altered in the disease. This can be regarded as *phenocopy*, a term originally coined by Goldschmidt in 1935 [4] to describe an individual whose phenotype, under a particular environmental condition, is identical to the one of another individual whose phenotype is determined by the genotype. In other words, in the phenocopy the environmental condition mimics the phenotype produced by a gene. In the case of ADRs, the environmental condition is represented by the exposure to the drug, whose effect mimics the phenotype (disease) produced by a gene in an individual. In this way, we can capitalize on all the knowledge about the genetic basis of diseases to explore mechanisms underlying ADRs.
Currently we consider two scenarios able to provide a causal inference of the signal (see Figure 1). First, we look for connections between the drug and the event through their associated protein profiles. Here, a connection is established if there are proteins in common between the drug-target and the

event-protein profile (Figure 1A). Many ADRs are caused by altered drug metabolism for which genetic variants in metabolizing enzymes are often responsible. Consequently, we also consider drug metabolism phenomena as an underlying mechanism of the observed ADR by assessing if the drug metabolites are targeting proteins that are known to be associated with the clinical event. Second, the association between the drug and the clinical event can involve proteins that are not directly associated with the drug and the clinical event, but indirectly in the context of biological networks. The final consequence of the drug action is the observed clinical event. Thus, the proteins in the drug-target profile and event-protein profile are mapped onto biological pathways to evaluate if the drug and the event can be connected through biological pathways (Figure 1B).

Implementation of the substantiation concept

The signal substantiation concept has been implemented by means of software modules that perform specific tasks of the processes. To allow access and integration of the modules in high-level analysis pipelines, the modules were implemented as web services and combined into data processing workflows to achieve the aforementioned signal substantiation. To standardize data exchanges between the different web services, we have developed two complementary schemas using XSD to define a common XML interoperability structure. The first one describes general data types[1] and the second one defines the specific types needed for signal filtering and substantiation in the context of the EU-ADR project[2]. Both schemas allow a smooth integration of the different modules in Taverna workflows, by enabling content and structure validation for the workflow input and output XML files. Moreover, the use of schemas facilitates further data transformations, for example, by applying XSL transformation to XML files of the signal substantiation workflow to create XGMML file graphs that can be visualized with Cytoscape. All workflows have been implemented and tested using Taverna Workflow Management system version 2.2.

Figure 1: The signal substantiation process involves the automatic search for evidences that support the causal inference of the potential signal. A. Signal substantiation through proteins. The profile of targets of the drug and its metabolites is obtained by in silico profiling methods (Drug-Target-Profile). The profile of proteins associated with the clinical event is obtained by mining DisGeNET (Event-Protein Profile). The profiles are compared to find proteins in common in both profiles (Drug-Event Linking Proteins). The evidences that support the association of the drug and event with the Drug-Event Linking proteins are explored to determine if they support the causal inference of the signal. B. Signal substantiation through pathways. Proteins in the Drug-Target-Profile and in the Event-Protein Profile are searched in The Human Protein Atlas database to determine if they are expressed in the same tissue and cell type. Proteins that share expression at both levels (tissue and cell type) are used to query Reactome database, and pathways that contain at least one protein from the Drug-Target-Profile and one protein from the Event-Protein Profile are retrieved. Then, these pathways are explored to determine if they support the causal inference of the signal.

---

[1] http://bioinformatics.ua.pt/euadr/common_types.xsd
[2] http://bioinformatics.ua.pt/euadr/euadr_types.xsd

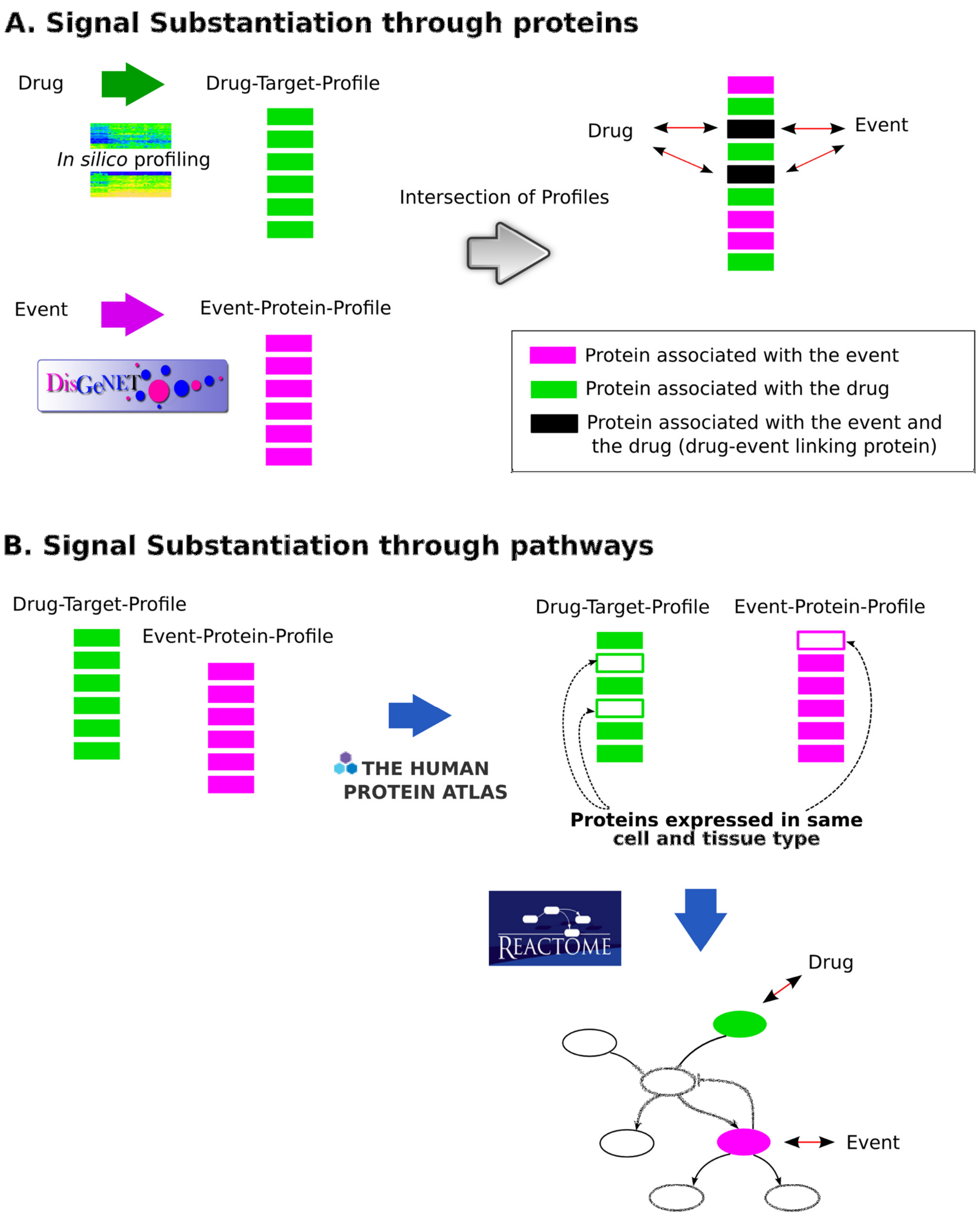
A. Signal Substantiation through proteins
Drug
Drug-Target-Profile
In silico profiling
Drug
Event
Intersection of Profiles
Event
Event-Protein-Profile
DisGeNET
Protein associated with the event
Protein associated with the drug
Protein associated with the event and the drug (drug-event linking protein)
B. Signal Substantiation through pathways
Drug-Target-Profile
Event-Protein-Profile
THE HUMAN PROTEIN ATLAS
Drug-Target-Profile
Event-Protein-Profile
Proteins expressed in same cell and tissue type
REACTOME
Drug
Event

getSmileFromATC (cglAlertService)

This method accepts as input a drug encoded by the ATC code at the 7-digits level and provides as output the chemical structure by means of SMILE (Simplified Molecular Input Line Entry Specification).

getUniprotListFromSmile (cglAlertService)

This method accepts as input a drug or metabolite encoded by a SMILE and returns a list of proteins that are related to the drug (Drug-Target-Profile). We use known drug-target associations and extend them with *in silico* target profiling methods [2]. Drug metabolites are obtained from a commercial database (GVK Biosciences) and are also processed by *in silico* target profiling. The evidences that support each drug-target relationship, such as the binding affinity of the compound to the protein or the source database, are provided.

getDiseaseAssociatedProteins (adrPathService)

This method accepts as input a clinical event (encoded as a list of UMLS® concept identifiers or as a string as defined in Table 1) and returns a list of proteins associated to the event (Event-Protein-Profile), by interrogating the DisGeNET database [3]. Evidences that support each association, including the association type, source database, publications discussing the association, and in the case of text-mining derived associations, the sentence that reports the gene-disease association, are provided.

getPathways (adrPathService)

This method assesses if proteins associated to the drug and the event are annotated to the same biological pathway by interrogating Reactome [5]. In general, pathway databases such as Reactome contain a canonical, general description of biological processes and pathways [6]. These pathways can be found in different cell types and tissues, or in different time points in the life of an organism; however, not all the pathway components might be active in all circumstances. Combining information from pathways with protein expression in tissues and cell types can result in a cell and tissue type specific view of a given pathway. Thus, this method combines annotation of proteins to pathways with information of protein expression in cells and tissues. Briefly, we determine if the proteins associated to the drug and the event are expressed in the same tissue and cell type according to the The Human Protein Atlas version 7.1 [7]. Only the proteins that share expression at both levels (tissue and cell type) are kept for the next step. Then, for this list of proteins, we retrieve all annotations to pathways using the Reactome web service (Figure 1B). The input of the method is composed of two lists of UniProt identifiers and the output is an XML document listing the pathways, the annotated proteins and their expression profile.

Workflow input: The substantiation workflow has five input ports, called *atc*, *event*, *eventType*, *eventName*, and *cytoscape*. The signal is represented by the ATC code of the drug at the 7-digits level (e.g. M01AH02 for celecoxib) and the event, which is defined by the three input ports *event*, *eventName* and *eventType*. We allow two different types of event definitions: events as defined in the EU-ADR project (Table 1), and events defined by a set of UMLS® concept identifiers. The input port *eventType* is then used to distinguish between the two definitions for events. The *eventName* can be set by the user and is only required for user-friendly visualization of the results. The *cytoscape* input port defines the location of the local Cytoscape installation (e.g. /home/user/cytoscape-v2.7.0); it is

optional and only required for the visualization of the signal substantiation results.

Workflow output: The output of the signal substantiation workflow consists of 7 ports representing different layers of the results. Besides the raw outputs from the individual web services (*drugTargetOutput* and *diseaseProteinOutput)*, the protein profile of the drug or its metabolites (*drugTargets*), and the protein profile of the event (*diseaseProteins*) are provided. The signal substantiation workflow combines two ways of connecting drug and event, through proteins or through biological pathways. The outcome of these results is shown to the user during workflow execution by pop-up windows. The list of connecting proteins, that is, the protein annotated to both the drug and the event is provided (*connectingProteins*). For a user-friendly visualization and analysis of the results, a Cytoscape graph (*CytoscapeResultGraph*) is generated. The graph is composed of three types of nodes: drug, event, and proteins, and two types of edges: drug-protein, protein-event. The attributes of the edges contain supporting information for each association, such as source databases, association type, binding value for the drug, etc. As result of the pathway analysis the output port *connectingPathways* provides a list of all pathways connecting drug and event that can be visualized as HTML file.

Workflow run:
The different web services run in parallel. The drug ATC code is first processed by the module getSmileFromATC, which returns the SMILE code of the drug. The SMILE code is then further processed by the module getUniprotListFromSmile, which returns the relationships between the drug and its targets, including targets of the metabolites of the drug. The event is processed by the module getDiseaseAssociatedProteins, which returns relationships between the event and associated proteins. The lists of proteins associated with drug or event are extracted by means of Java scripts using XPath queries and are further processed to remove duplicates. The module ConvertToCytoscapeGraph converts the output of the web services to a Cytoscape graph for user-friendly visualization by means of XSL transformation. For the signal substantiation through proteins, the two protein profiles are combined to determine the proteins in common between the two profiles (module CheckIntersection). For the signal substantiation through pathways, the two protein profiles are subjected to the module getPathways, which returns a list of pathways to which at least one drug and one event protein that are expressed in the same tissue are annotated to. The output is further processed by module ConvertToHTML, which generates an HTML file listing the pathways that connect the drug and the event.

3. License

The ADR substantiation workflow is distributed under the GNU GENERAL PUBLIC LICENSE version 3 (http://www.gnu.org/licenses/gpl.html)

4. Requirements

The workflow was developed and tested in Taverna workbench 2.2. To visualize the results as a graph, you will need Cytoscape. You can use Cytoscape versions 2.7 or 2.8

a. Install Taverna

Download Taverna workbench 2.2
http://www.taverna.org.uk/download/workbench/2-2/
Follow the instructions provided for installation and launch.

b. Install Cytoscape

Cytoscape version 2.7 or version 2.8 can be downloaded from http://www.cytoscape.org/
To install, follow the instructions according to your platform as explained in the Cytoscape web page.
For more information about Cytoscape functionalities check the Cytoscape user manual corresponding to the Cytoscape version you are using.

5. How to run the workflow

a. Launch Taverna

b. Open the workflow

Open the workflow file ADR_substantiation.t2flow
A pop-up window showing the information on the https certificate will pop-up, click on *Trust Once* or *Trust Always* to continue (Figure 2).

Figure 2

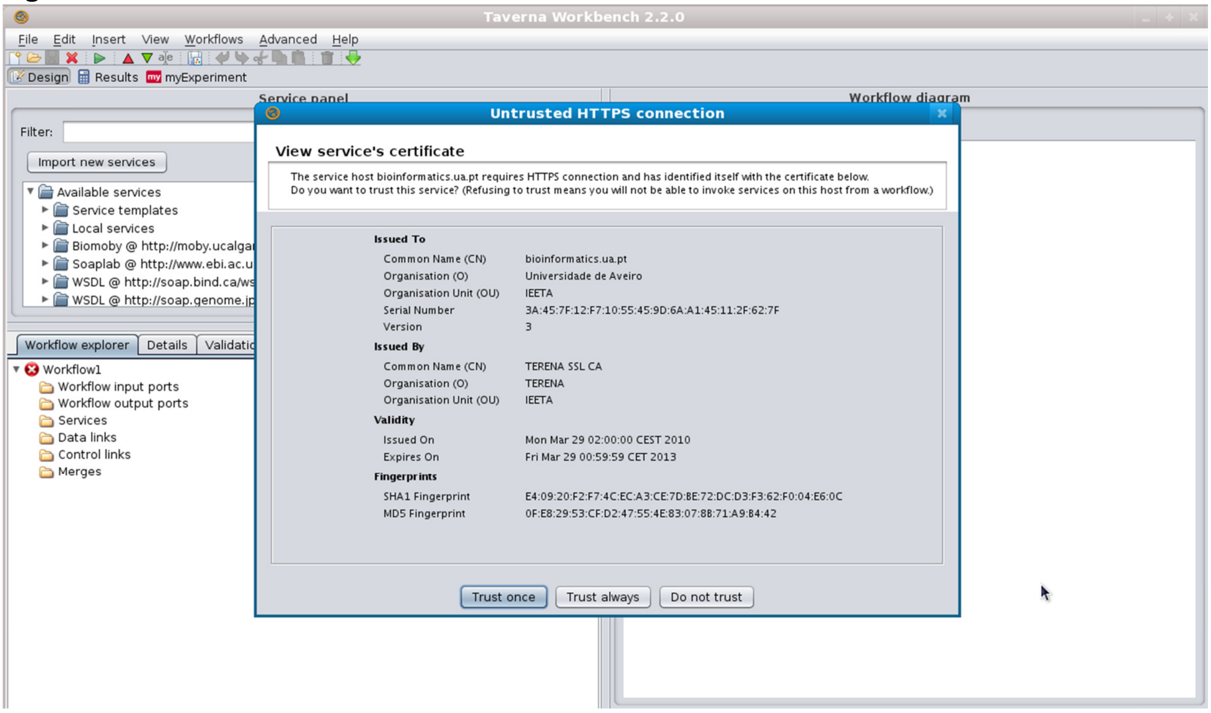

The workflow will load. You can inspect the structure of the workflow in the Workflow Diagram Panel (Figure 3).

Figure 3

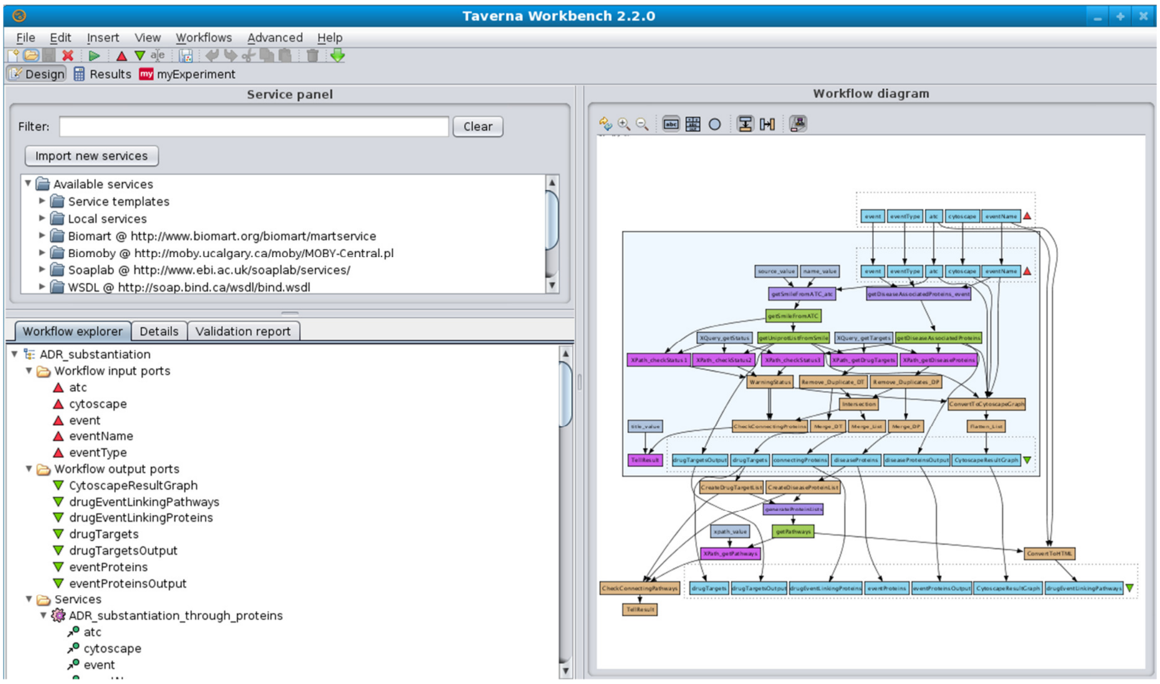

c. Run the workflow

Go to *File --> Run workflow...* or use the *Run workflow* shortcut button in Taverna.

Before running a workflow, Taverna performs a validation of the workflow. You will see a pop-up window indicating that the workflow has warnings (Figure 4), you can ignore them and press *yes* to proceed.

Then, a pop-up window with the input values required to run the workflow will appear (Figure 5).

Figure 4

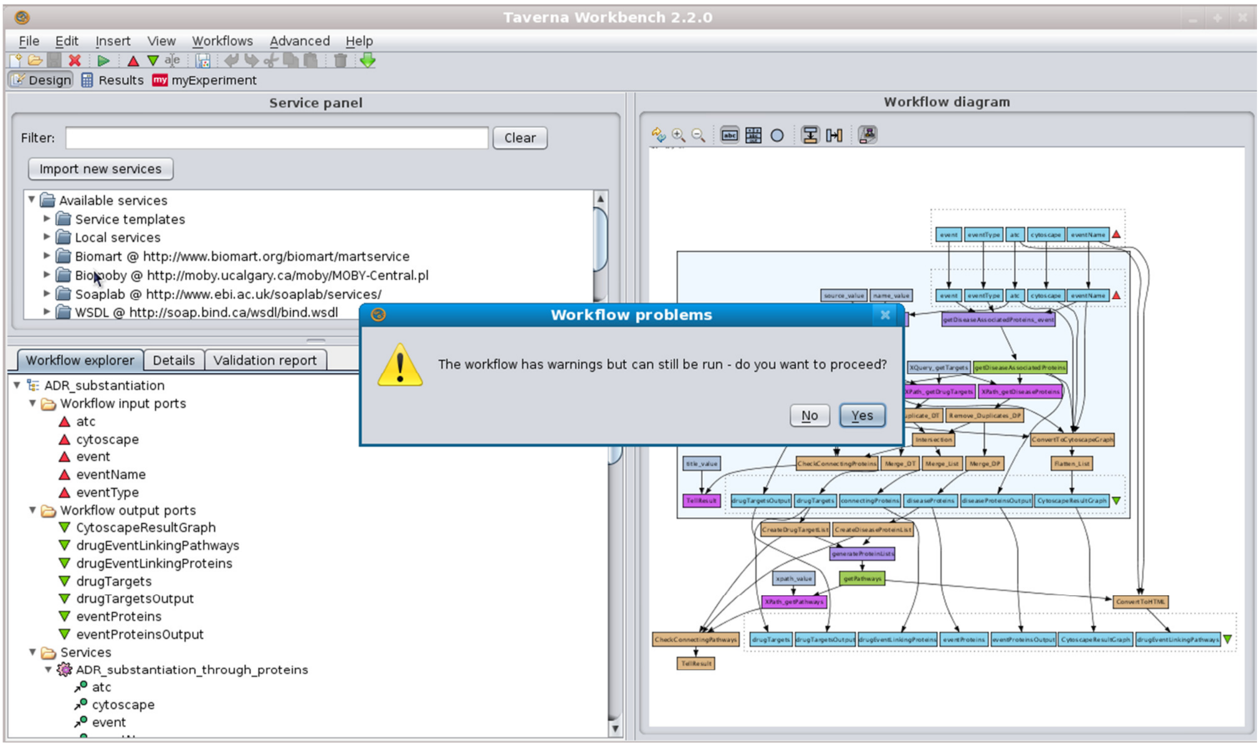

Figure 5

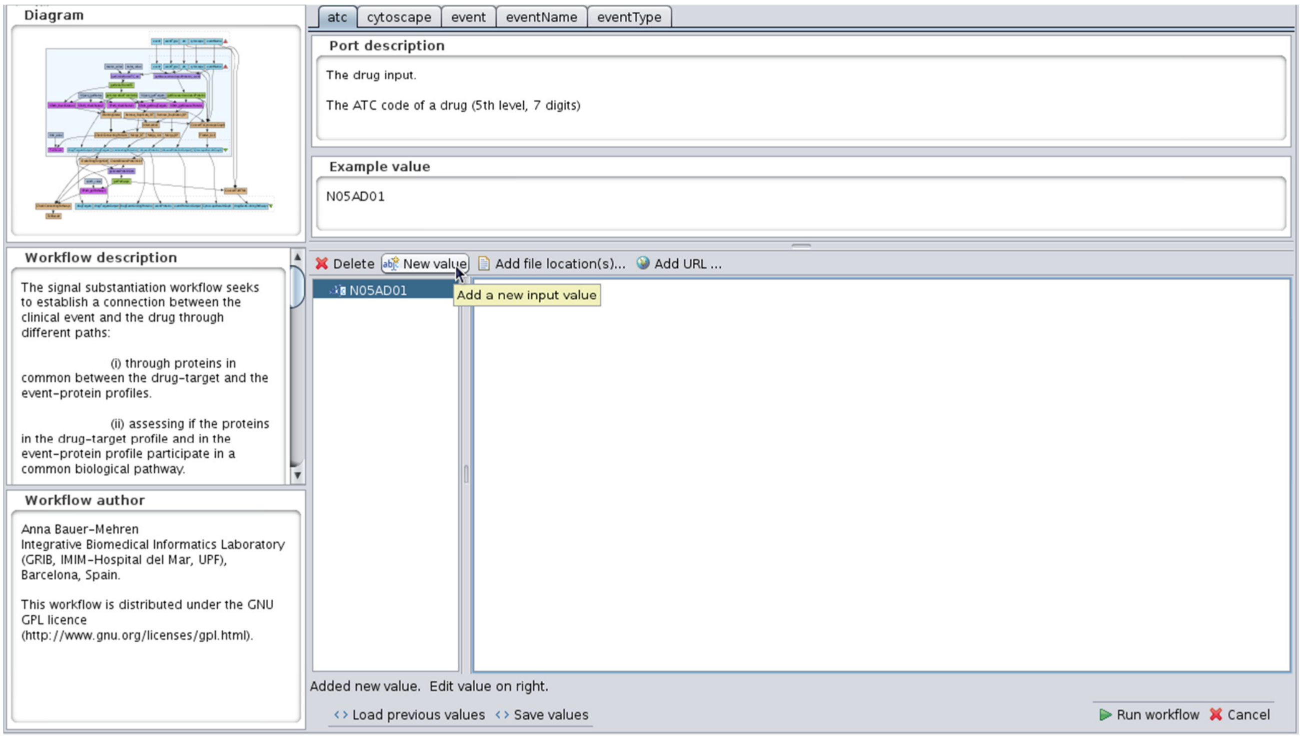

The ADR-S workflow has the following values as input:

- *atc*: corresponds to the input drug. It accepts an ATC (Anatomical Therapeutic Chemical, http://www.whocc.no/atc_ddd_index/) code for a drug (5th level, 7 digits). Example value: N05AD01 (Figure 5) encoding the antipsychotic drug haloperidol.

- *event: corresponds to the input clinical event. For the clinical events, the following input types are allowed:*
  1) UMLS: UMLS concept identifiers, for example: C0003811
  2) EUADR_EVENT: clinical events observed as adverse drug reactions according to the EU-ADR project, for example UGIB. See section 6 for more details

  If you use option 1), insert here a single UMLS concept identifier or a list of identifiers (Figure 6).

  If you use option 2), insert here the name of the EUADR_EVENT as defined in section 6.

Figure 6

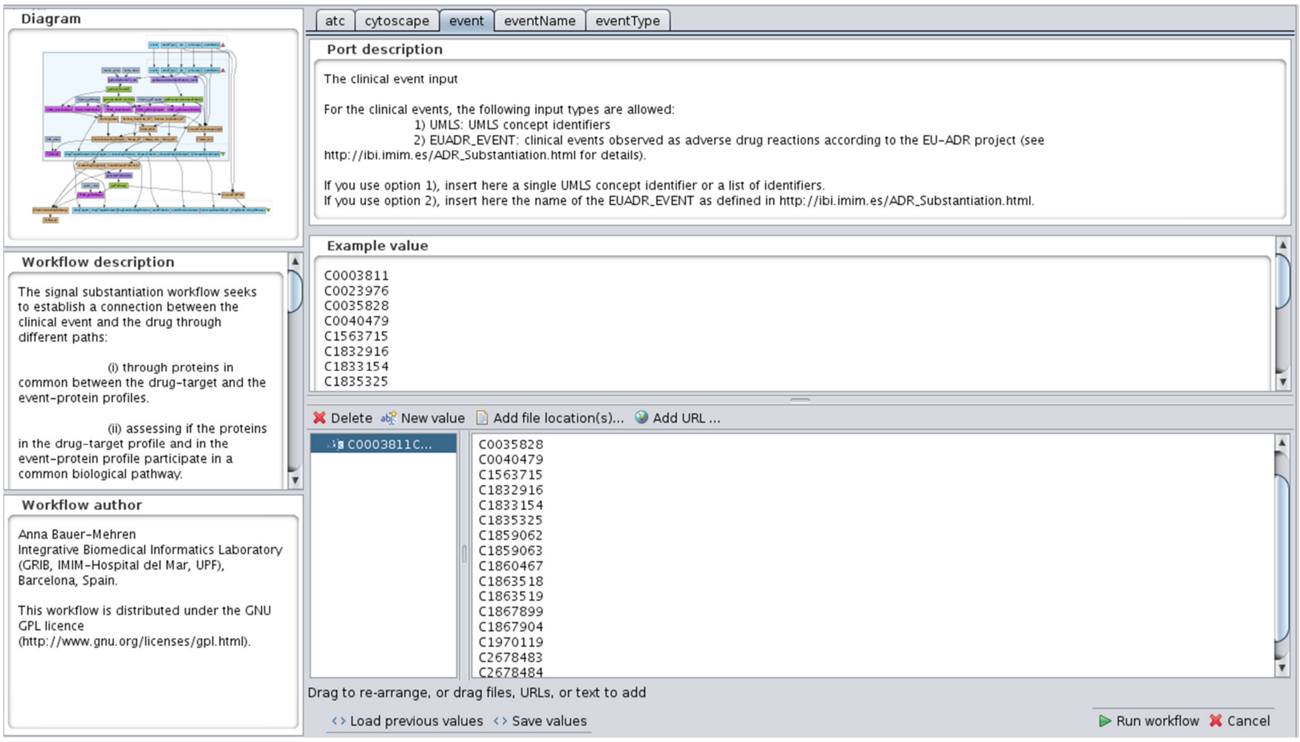

- *eventType: Type of the event to be processed.*
For the clinical events, the following types are allowed:
  1) UMLS: if you use UMLS concept identifiers (single identifier or a list of identifiers)
  2) EUADR_EVENT: if you use clinical events as defined in the EU-ADR project (see section 6)

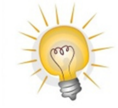

Attention, the eventType is CASE SENSITIVE!!

- *eventName: use this option to define a name for the clinical event. This is required for user-friendly visualization of the results. (Figure 7). Example: long QT syndrome*

Figure 7

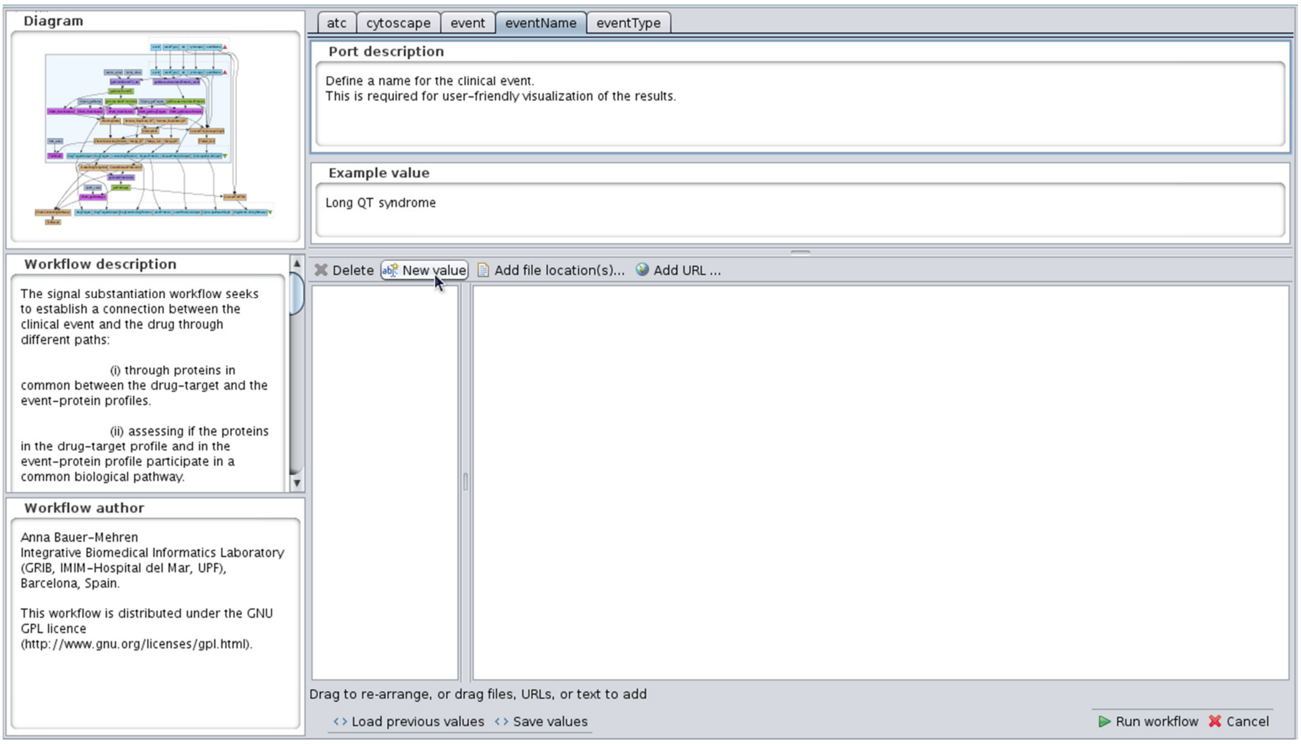

- *cytoscape*: indicate the path of your Cytoscape installation (Figure 8). Example: /home/laura/Cytoscape_v2.7.0/
- Once you have specified all the input values, click *Run workflow*!
- For this example we will use the default values provided by the workflow, which represent the association of **haloperidol** (N05AD01) with a list of UMLS concept identifiers representing the clinical event **prolongation of QT interval**.
- You will be prompted to Results panel where you can monitor the progress of the workflow run (Figure 9).

Figure 8

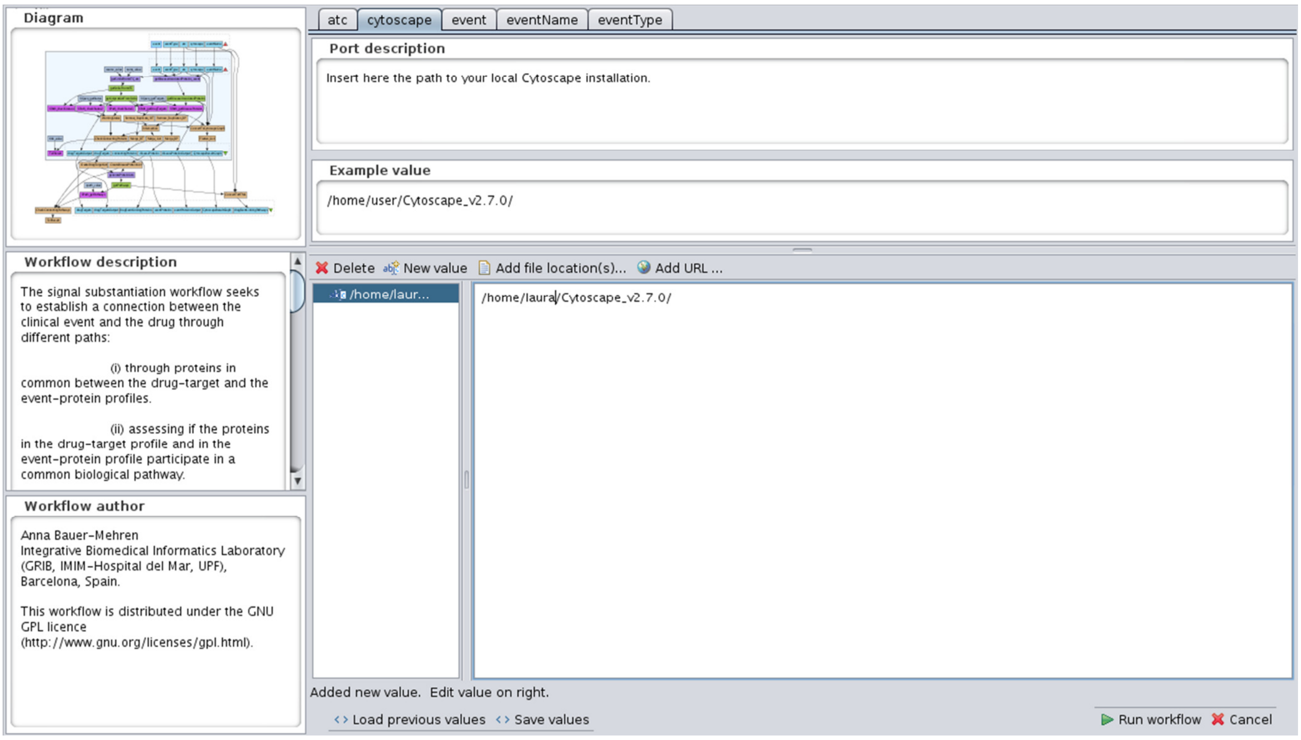

Figure 9

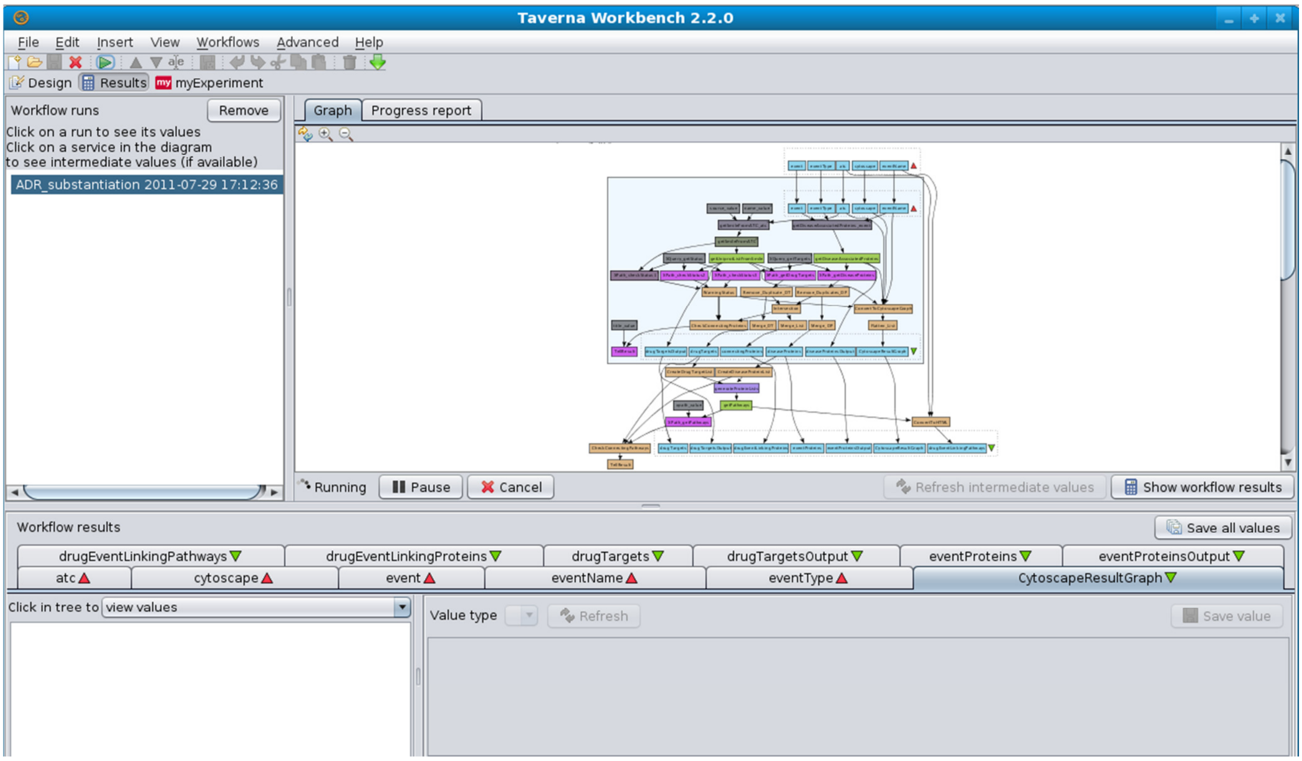

d. Workflow results

When the first part of the workflow execution finishes, a pop-up window will appear indicating the results (Figure 10).

Figure 10

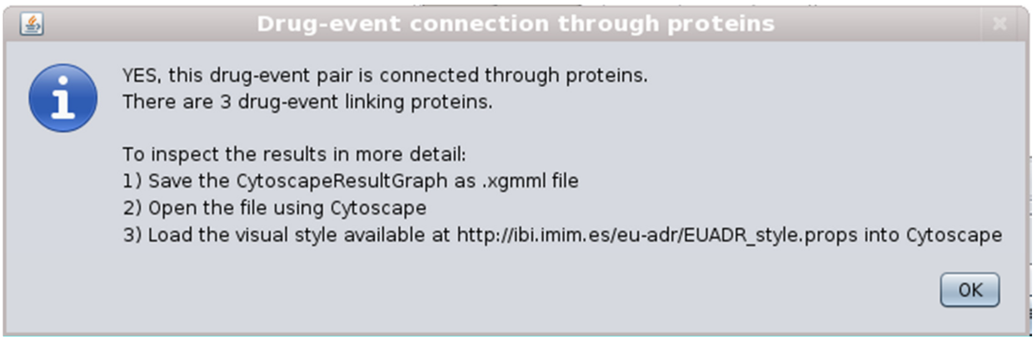

When the second part of the workflow execution finishes, a pop-up window will appear indicating the results (Figure 11). Once the workflow execution finishes, all results are found in the Taverna results panel (Figure 12).

Figure 11

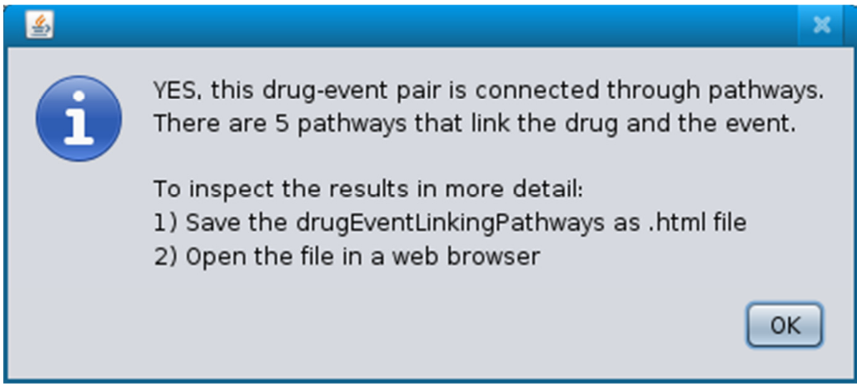

Cytoscape graph results

If you provided the path of your local Cytoscape installation, and the workflow generated results on the drug targets and the event proteins, the outcome will be displayed as a Cytoscape graph. Cytoscape will launch automatically load the Cytoscape graph file (Figure 13). Green nodes represent Drug or Metabolite, pink nodes represent the Event and blue nodes represent Protein. Node and Edge attributes are described in Tables 2 and 3. Figure 13 displays the Cytoscape graph using the Organic Layout found in the Cytoscape function *Layout* → *yFiles* → *Organic.*

Figure 12

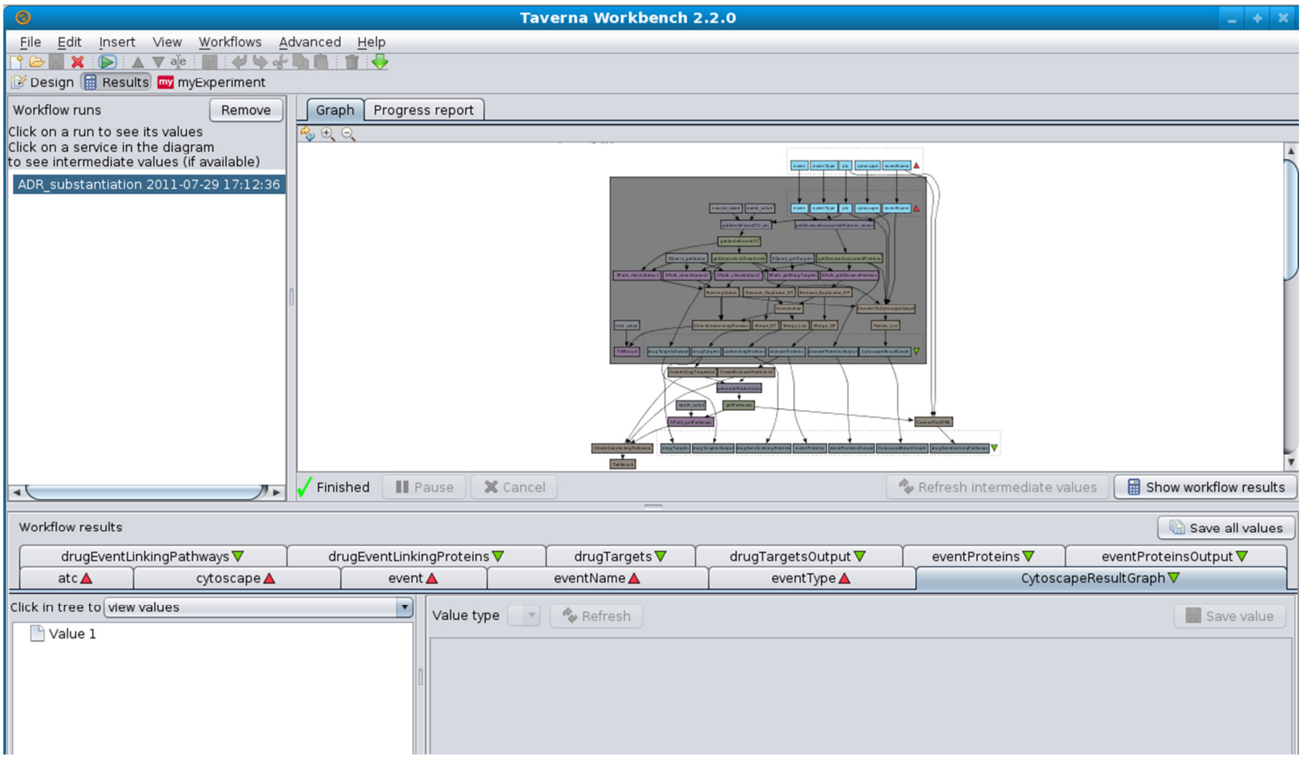

Figure 13

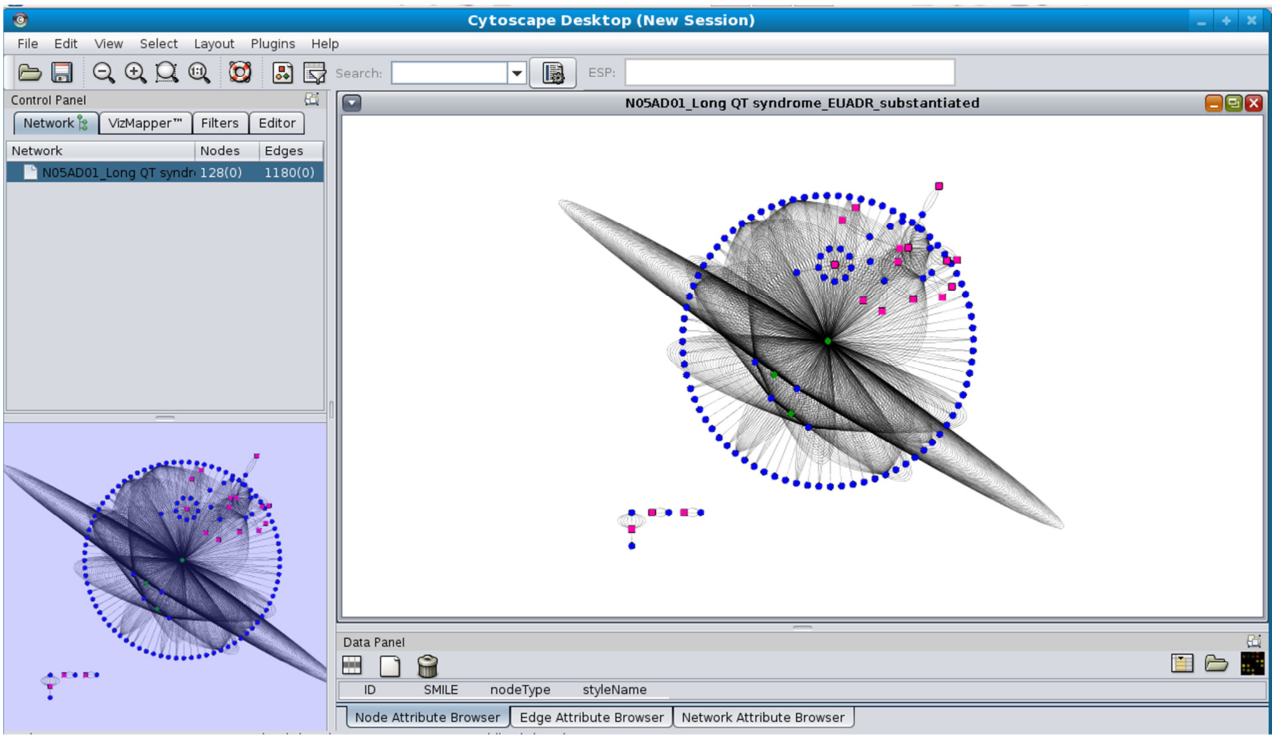

To find out if the drug and the event are connected through proteins, you can use Cytoscape functionalities. The following steps will guide you to use Cytoscape functions to select nodes that link the drug and the event nodes (Protein linking nodes).

1. Select the proteins nodes that constitute the Drug-Target-Profile.
   a. Using the *nodeType* attribute drug, select the First neighbours of the drug nodes, using the menu *Select* → *Nodes* → *First Neighbours of selected nodes*.
   b. Create a new graph with the selected nodes: *File* → *New* → *Network* → *From selected nodes, all edges*. This will create a new sub-graph representing the Drug-Target-Profile.
2. Select the proteins nodes that constitute the Event-Protein-Profile.
   a. Repeat the same procedure to create a graph representing the Event-Protein-Profile.
3. Now we will find the intersection between the Drug-Target-Profile and the Event-Protein-Profile, this intersection will represent the drug-event linking proteins.
   a. Go to *Plugins* → *Advance Network Merge*
   b. Select operation *intersection*
   c. Select the networks you want to merge
   d. In the Advanced Network Merge panel, choose the attributes of the nodes you will use to merge, in this case choose ID.
   e. By clicking *Merge* you will obtain the protein nodes that link the drug and the event. In the example using haloperidol and prolongation of QT interval, this operation will result in 3 protein nodes (KCNH1, KCNH2, CACNA1C).

You can inspect node and edge attributes to learn more about the connections between the drug and the event through proteins.

If the drug and the event are no connected through proteins, this operation will lead to an empty set.

Alternatively, you can store the results as a Cytoscape XGMML file. Go to the CytoscapeResultGraph and save the Value as XGMML file.

To inspect the results later, follow these steps:

1. Open Cytoscape
2. Load the XGMML file in Cytoscape: Go *to Import* → *Network (Multiple Fyle Types) ….* and select the XGMML file
3. Change the layout to Organic to explore the network: Got to *Layout* → *yFiles* → *Organic*
4. If required, load the visual style located in this URL to have a nice visualization of the results: http://ibi.imim.es/eu-adr/EUADR_cytoscape.props

Pathway results

To visualize the results of the Pathway analysis, go to the drugEventLinkingPathways tab, and save the Value as an html file. You can inspect the results in any web browser.

e. Invalid Input values

If you enter an invalid string for the drug, you will get the following message:

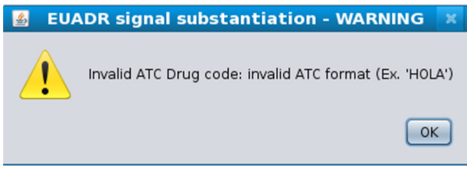

Alternatively, if you enter an invalid string for the event, you will get the following error message:

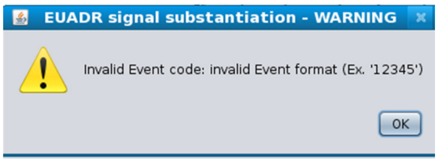

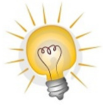

Attention, the *eventType* parameter is CASE SENSITIVE!!

6. EU-ADR events

The EU-ADR project focuses on a selection of adverse drug reactions that are monitored in electronic health records and further analyzed by the filtering and substantiation workflows [8,9]. These events were defined in terms of UMLS Metathesaurus® concept identifiers as described in [8,10]. The event codes and names as defined in the EU-ADR project are listed in Table 1. The mapping of events codes or strings to UMLS Metathesaurus® concept identifiers and other vocabularies such MeSH® and OMIM is implemented within the web services. The ADR-S workflow accepts events as defined in the EU-ADR project or any other clinical event defined by UMLS concept identifier. The UMLS concept identifiers are processed to map them to MeSH® and OMIM identifiers using UMLS Metathesaurus®.

7. Service ports

http://ibi.imim.es/axis2/services/AdrPathService?wsdl
http://cgl.imim.es/axis2/services/cglAlertService?wsdl

8. Workflow URL

http://www.myexperiment.org/workflows/1988.html

9. References

1. Swanson DR. (1986) Fish oil, raynaud's syndrome, and undiscovered public knowledge. Perspect Biol Med 30(1): 7-18.

2. Garcia-Serna R, Mestres J. (2010) Anticipating drug side effects by comparative pharmacology. Expert Opin Drug Metab Toxicol 6(10): 1253-1263.

3. Bauer-Mehren A, Rautschka M, Sanz F, Furlong LI. (2010) DisGeNET: A cytoscape plugin to visualize, integrate, search and analyze gene-disease networks. Bioinformatics 26(22): 2924-2926.

4. Lenz W. (1970) Phenocopy. Hum Genet 9(3): 227-229.

5. Vastrik I, D/'Eustachio P, Schmidt E, Joshi-Tope G, Gopinath G, et al. (2007) Reactome: A knowledge base of biologic pathways and processes. Genome Biol 8: R39.

6. Bauer-Mehren A, Furlong LI, Sanz F. (2009) Pathway databases and tools for their exploitation: Benefits, current limitations and challenges. Mol Syst Biol 5: 290.

7. Uhlen M, Oksvold P, Fagerberg L, Lundberg E, Jonasson K, et al. (2010) Towards a knowledge-based human protein atlas. Nat Biotechnol 28(12): 1248-1250.

8. Trifiro G, Pariente A, Coloma PM, Kors JA, Polimeni G, et al. (2009) Data mining on electronic health record databases for signal detection in pharmacovigilance: Which events to monitor? Pharmacoepidemiol Drug Saf 18(12): 1176-1184.

9. Coloma PM, Schuemie MJ, Trifiro G, Gini R, Herings R, et al. (2011) Combining electronic healthcare databases in europe to allow for large-scale drug safety monitoring: The EU-ADR project. Pharmacoepidemiol Drug Saf 20(1): 1-11.

10. Avillach P, Mougin F, Joubert M, Thiessard F, Pariente A, et al. (2009) A semantic approach for the homogeneous identification of events in eight patient databases: A contribution to the european eu-ADR project. Stud Health Technol Inform 150: 190-194.

10. Tables

Table 1

Event code	Event name
BE	Bullous Eruptions
AS	Anaphylactic Shock
ARF	Acute Renal Failure
AMI	Acute Myocardial Infarction
ALI	Acute Liver Injury
CARDFIB	Cardiac Valve Fibrosis
UGIB	Upper gastrointestinal bleeding
RHABD	Rhabdomyolysis
PANCYTOP	Aplastic anemia/Pancytopenia
NEUTROP	Neutropenia/Agranulocytosis
QTPROL	QT Prolongation

Table 2: Node attributes in the Cytoscape graph

Entity	ID	SMILE	styleName	nodeType
**Drug**	Internal identifier for the node in the network. The ATC code for the drug.	The SMILE string corresponding to the drug structure.	Common name for the node. The generic drug name.	Drug
**Metabolite**	Internal identifier for the node in the network. Internal identifier for the metabolite.	Not provided	Common name for the node. Numbered metabolite.	Drug
**Event**	Internal identifier for the node in the network. The UMLS® CUI for the event.	Not applicable	Common name for the node. Name of the UMLS® CUI concept extracted from UMLS®.	Event
**Protein**	Internal identifier for the node in the network. The UniProt accession number for the protein.	Not applicable	Common name for the node Gene symbol for the protein as in UniProt.	Protein

Table 3: Edge attributes in the Cytoscape result graph

	ID	bindingValue	evidenceLink	evidenceSource	evidenceType	relationshipType
**Drug-protein**	Internal identifier constructed of the ATC code of the drug and the UniProt identifier of the protein.	The binding affinity value as reported in the original database.	Not applicable	Database providing the association.	OBSERVATIONAL for associations taken from databases. SIMILARITY for associations from *in silico* profiling.	BINDS for drug-target binding
**Metabolite-protein**	Internal identifier constructed of the metabolite identifier and the UniProt identifier for the protein.	The binding affinity value as reported in the original database or transferred during *in silico* profiling.	Not applicable	Database providing the association.	OBSERVATIONAL for associations taken from databases. SIMILARITY for associations from *in silico* profiling.	BINDS for metabolite-target binding.
**Event-protein**	Internal identifier constructed of the UMLS® CUI concept and the UniProt identifier of the protein.	Not applicable	PubMed identifier of the publication supporting the association, empty if not available.	Database providing the association.	OBSERVATIONAL for associations from curated databases. TEXT-MINING for text-mining derived associations.	Association type according to the gene-disease association ontology available in [3].

11. Funding

This work was supported by the European Commission [EU-ADR, ICT-215847], Innovative Medicines Initiative [eTOX,115002], the AGAUR [to A.B.M.], Instituto de Salud Carlos III FEDER (CP10/00524) and COMBIOMED grants. The Research Unit on Biomedical Informatics (GRIB) is a node of the Spanish National Institute of Bioinformatics (INB). The authors wish to thank the NLM® for making UMLS® and MesH® available free of charge.

12. Information about this document

Creative Commons License

ADR substantiation workflow tutorial by Laura I. Furlong is licensed under a Creative Commons Attribution-ShareAlike 3.0 Unported License.
